# Inhibition of anti-apoptotic Bcl-2 family members promotes synergistic cell death with ER stress inducers by disrupting autophagy in glioblastoma

**DOI:** 10.1038/s41420-025-02632-4

**Published:** 2025-07-24

**Authors:** Tianyi Huang, Satoshi Takagi, Sumie Koike, Ryohei Katayama

**Affiliations:** 1 Division of Experimental Chemotherapy, Cancer Chemotherapy Center, Japanese Foundation for Cancer Research, Tokyo, Japan; 2 Department of Computational Biology and Medical Sciences, Graduate School of Frontier Sciences, the University of Tokyo, Tokyo, Japan

**Keywords:** CNS cancer, Autophagy, Apoptosis, Stress signalling

## Abstract

Glioblastoma (GBM) remains one of the most aggressive and challenging brain tumors. Unfortunately, current clinical treatment options offer limited efficacy, highlighting the necessity for uncovering novel therapeutic strategies. Here, monotherapy and combination library screening were employed, and identified that the efficacy of obatoclax, a pan-Bcl-2 family inhibitor, was improved significantly when combined with ER-stress inducers, including tunicamycin. Combinatorial knockdown of anti-apoptotic proteins confirmed that the loss of Mcl-1 and Bcl-xL synergistically enhanced apoptosis under ER stress conditions. Although ER stress inducers triggered the stress response in GBM cells, obatoclax co-treatment enhanced this response by upregulating ATF-4 and CHOP, which promoted apoptosis along with increased caspase 3/7 activity and cleavage of PARP. ATF-4 knockdown significantly decreased the apoptosis induced by obatoclax and tunicamycin co-treatment and reduced the expression of CHOP and BIM. Under ER stress responses, GBM cells exerted an autophagy response to recover from the stress condition; however, obatoclax co-treatment disrupted the autophagy responses, particularly by disrupting autophagic cargo degradation. Our findings suggest that targeting Mcl-1 and Bcl-xL, coupled with ER-stress induction, could be a promising strategy for the treatment of GBM, highlighting the potential for combination therapies involving pan-Bcl-2 family inhibitors to overcome current limitations in the treatment of GBM.

## Introduction

Glioblastoma (GBM), the predominant malignant primary brain tumor, accounts for 51.5% of all primary malignant brain and central nervous system tumors throughout the United States [1]. Despite integrating surgery, chemotherapy, and radiotherapy for the primary treatment of GBM, the median survival of patients with GBM remains grim at <12 months [2]. Such a poor prognosis is exacerbated by the limited availability of FDA-approved first-line chemotherapy drugs, with only temozolomide having been approved since 2005 [3]. Moreover, some patients respond unfavorably to standard therapeutic approaches, leading to dishearteningly low overall survival rates, frequently marred by recurrences [4, 5]. Considering the limited availability of therapies for GBM, uncovering innovative and effective treatment strategies is critical for improved clinical outcomes.

The B-cell lymphoma 2 (Bcl-2) family plays a pivotal role in regulating cell apoptosis. Based on their functions and sequence homology, this protein family can be categorized into two classes: anti-apoptotic proteins (e.g., Bcl-2, Bcl-xL, Mcl-1, Bcl-w, and Bfl-1) and pro-apoptotic proteins (e.g., multiple domain pro-apoptotic effectors and the single domain BH3-only proteins) [6, 7]. Small-molecule antagonists of the anti-apoptotic Bcl-2 family proteins have seen significant development [8]. The pioneering Bcl-2 family inhibitor, ABT-737, exhibits high affinity binding to BCL-2, BCL-XL, and BCL-W, whereas its orally bioavailable analog, navitoclax (ABT-263), was capable of suppressing certain solid tumors [9, 10]. Simultaneously, venetoclax (ABT-199), a selective Bcl-2 inhibitor, had been approved for the treatment of hematologic malignancies, including acute myeloid leukemia [11, 12].

Recent studies have reported that Mcl-1 and Bcl-xL, anti-apoptotic members of the Bcl-2 family, play essential roles in the survival of the developing nervous system [13]. Obatoclax, also known as GX015-070, is a pan-Bcl-2 family inhibitor capable of binding to the hydrophobic groove of anti-apoptotic members and counteracting their function [14, 15]. In particular, obatoclax is highly efficient in neutralizing Mcl-1 [16] given its unique ability to co-target Mcl-1, Bcl-xL, and Bcl-2 [17, 18]. A phase I clinical trial has indicated that obatoclax can cross the blood–brain barrier [19]. Studies have also indicated that obatoclax can potently induce cell death in certain tumor types, such as myeloma, leukemia, and non-small-cell lung cancer [20–24]. Studies have reported that dual mTORC1/2 inhibitors in atypical teratoid/rhabdoid tumors sensitized cells to exogenous stress induced by obatoclax, with a significant synergistic effect having been observed with combination therapy [25]. Although the mechanism underlying the synergy between obatoclax and other agents remains unclear, researchers have found that many of such synthetic lethal outcomes were related to apoptosis or cell stress [26–29].

Endoplasmic reticulum (ER) stress is a cellular condition characterized by the inability of the ER to fold and modify proteins properly. Recent studies have suggested that activating IRE1α, JNK1, and calcium signaling in the unfolded protein response (UPR) pathway can trigger ER calcium efflux and cause cell death in GBM [30]. Long-term simvastatin-induced UPR has been reported to overcome resistance and sensitize GBM cells to temozolomide therapy [31]. However, canceling low-level chronic UPR can resensitize GBM cells to temozolomide, indicating that ER stress and UPR can either protect or kill tumor cells depending on their severity [32]. Tunicamycin, a natural antibiotic produced by *Streptomyces* strains, has been widely used for understanding the interaction between tumor survival and ER-stress response [33–35]. It inhibits N-linked glycosylation during protein synthesis, causing unfolded proteins to accumulate within the ER and activate the UPR [36].

Studies show that inducing ER stress promotes autophagy in certain tumor cells [37, 38], with obatoclax also being reported to be involved in autophagy regulation. Although obatoclax accumulating at the lysosome was capable of disturbing autolysosome formation and autophagy in several types of cancer cells [39–41], mitochondrial stress-mediated autophagy induction was also observed following obatoclax treatment, indicating its ability to induce autophagy in certain tumor cells, such as human oral cancer and GBM [42, 43].

Here, our study employed inhibitor library screening to identify potential therapies for GBM, subsequently uncovering a model wherein obatoclax disrupts tunicamycin-induced autophagy, thereby further enhancing the level of ER stress. Aggravated ER stress synergizes with the inhibition of Mcl-1/Bcl-xL/Bcl-2, which enhances cell death in GBM. These results revealed a unique lethal regulation mediated by mitochondrial apoptosis, ER stress, and autophagy, providing potential insights into the development of novel therapeutic strategies involving the targeting of Bcl-2 families for GBM treatment.

## Results

### Pan-Bcl-2 family inhibitor obatoclax, but not navitoclax, reduced the GBM cell viability

**Fig. 1:**
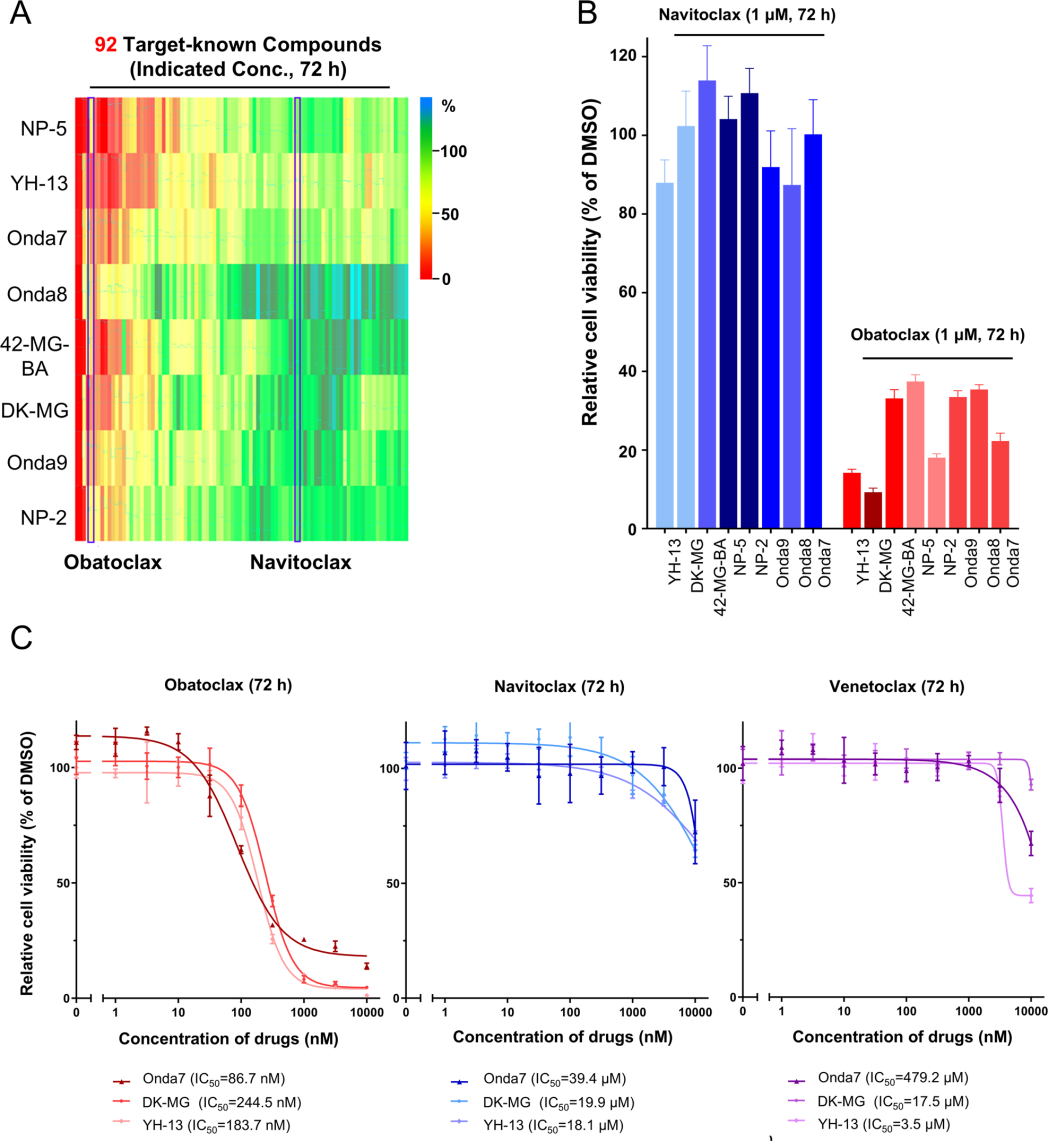
Exclusive inhibition of GBM cell lines induced via the pan-Bcl-2 inhibitor obatoclax. Cell viability was measured using the CellTiter-Glo assay after 72 h of treatment and presented as mean ± SD. **A** Heatmap of inhibitor library screening on 8 GBM cell lines with 92 compounds with known targets. The cell viability in non-treated (DMSO) controls was calculated as 100%. Treatment time was 72 h with concentrations indicated in Supplementary Table 1. **B** Comparison of sensitivity to 1 µM of obatoclax and navitoclax in 8 GBM cell lines after 72 h (*n* **=** 6). **C** Comparison of dose–response curves of 72 h treatment for obatoclax, navitoclax, and venetoclax in the Onda7, DK-MG, and YH-13 cell lines (*n* **=** 3).

To identify potential molecular targets for GBM treatment, eight different GBM cell lines (Onda7, Onda8, Onda9, NP-2, NP-5, 42-MG-BA, DK-MG, and YH-13) were treated with our in-house library containing 92 compounds with known targets. Cell survival rates after 72 h of treatment revealed significant variability in drug sensitivity among the cell lines. Obatoclax demonstrated high growth inhibitory effects on all eight GBM cell lines (Fig. 1A and Fig. S1A). In contrast, navitoclax promoted minimal growth inhibition across all eight GBM cell lines. Further analysis of cell survival rates following treatment with 1 μM of obatoclax and navitoclax confirmed significant differences in drug sensitivity (Fig. 1B). Serially diluted treatment of highly sensitive GBM cell lines (Onda7, DK-MG, and YH-13) with different Bcl-2 family inhibitors revealed that obatoclax exhibited significantly higher growth inhibitory effects than did navitoclax and venetoclax (Fig. 1C). Given navitoclax’s high selectivity for Bcl-2 and Bcl-xL and venetoclax’s high selectivity for Bcl-2, these results highlight the importance of Mcl-1 in GBM cell survival.

### Combinatorial knockdown of Mcl-1 and Bcl-xL inhibits growth and induces apoptosis of GBM cells

**Fig. 2:**
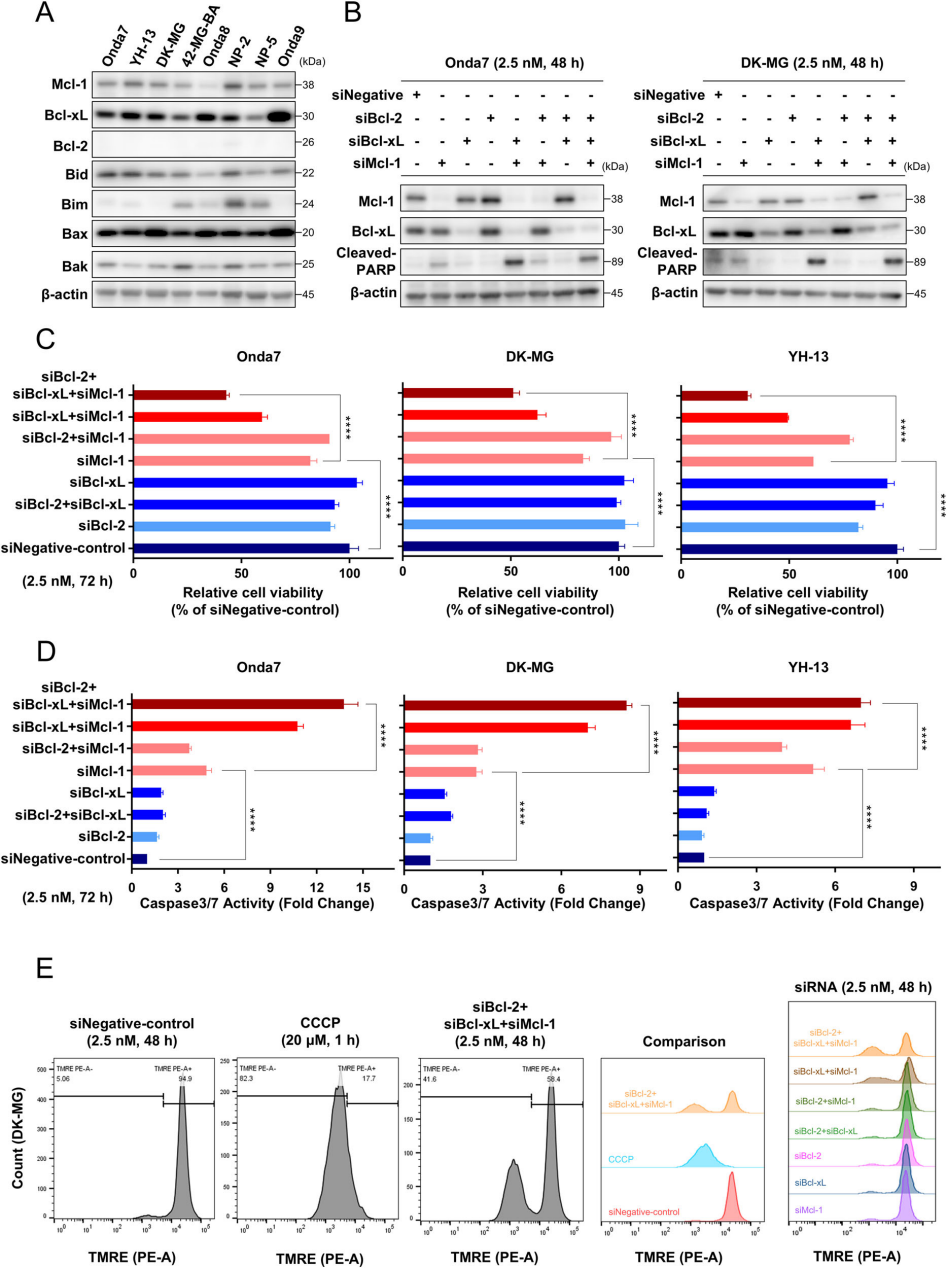
Targeting of Mcl-1 and Bcl-xL exerted therapeutic potential against GBM cell lines. **A** Bcl-2 family protein expression in 8 GBM cell lines were detected via Western blotting. **B** Mcl-1, Bcl-xL, and cleavage of PARP were detected via Western blotting 48 h after treatment with 2.5 nM siNegative, siMcl-1, siBcl-xL, or siBcl-2. Bcl-2 was not detectable due to low expression. **C** Cell viability of Onda7, DK-MG, and YH-13 was measured using the CellTiter-Glo assay 72 h after treatment with 2.5 nM siNegative, siMcl-1, siBcl-xL, or siBcl-2 and presented as mean ± SD (*n* = 6). ****P < 0.0001. **D** Caspase 3/7 activity of Onda7, DK-MG, and YH-13 were measured using the Caspase-Glo 3/7 assay 72 h after treatment with 2.5 nM siNegative, siMcl-1, siBcl-xL, or siBcl-2 and presented as mean fold change ± SD (*n* = 6). ****P < 0.0001. **E** Mitochondrial membrane potential of DK-MG cells was measured via TMRE fluorescence by flow cytometry after 48 h treatment of 2.5 nM siNegative, siMcl-1, siBcl-xL, or siBcl-2, or after 1 h treatment of 20 µM carbonyl cyanide 3-chlorophenylhydrazone (CCCP) as a positive control for mitochondrial depolarization.

Expression levels of Bcl-2 family members in GBM cells were investigated using Western blotting (Fig. 2A) to clarify the patterns of various apoptosis-related proteins. Our results suggested that Mcl-1 and Bcl-xL, but not Bcl-2, were dominantly expressed among GBM cell lines, potentially further influencing the sensitivity to obatoclax and navitoclax.

To assess the roles of Mcl-1 and Bcl-xL in GBM survival, siRNA knockdown was performed on three obatoclax-sensitive GBM cell lines. Combinatorial knockdown of Mcl-1 and Bcl-xL resulted in elevated cleaved PARP production, indicating potent apoptosis induction (Fig. 2B). Although Bcl-xL or Bcl-2 knockdown alone had minimal impact on cell growth or apoptosis, Mcl-1 knockdown significantly reduced cell viability and induced apoptosis, with the combined knockdown of Mcl-1 and Bcl-xL showing even greater effects (Fig. 2C, D). Depolarization of mitochondrial membrane potential was also observed when Mcl-1 and Bcl-xL were knocked down simultaneously, indicating potential mitochondrial dysfunction (Fig. 2E and Fig. S2A). These findings suggest that Mcl-1 and Bcl-xL are key genes in GBM survival, underscoring their potential as therapeutic targets in GBM treatment.

### Obatoclax-induced growth inhibition is enhanced by tunicamycin, an endoplasmic reticulum stress inducer

**Fig. 3:**
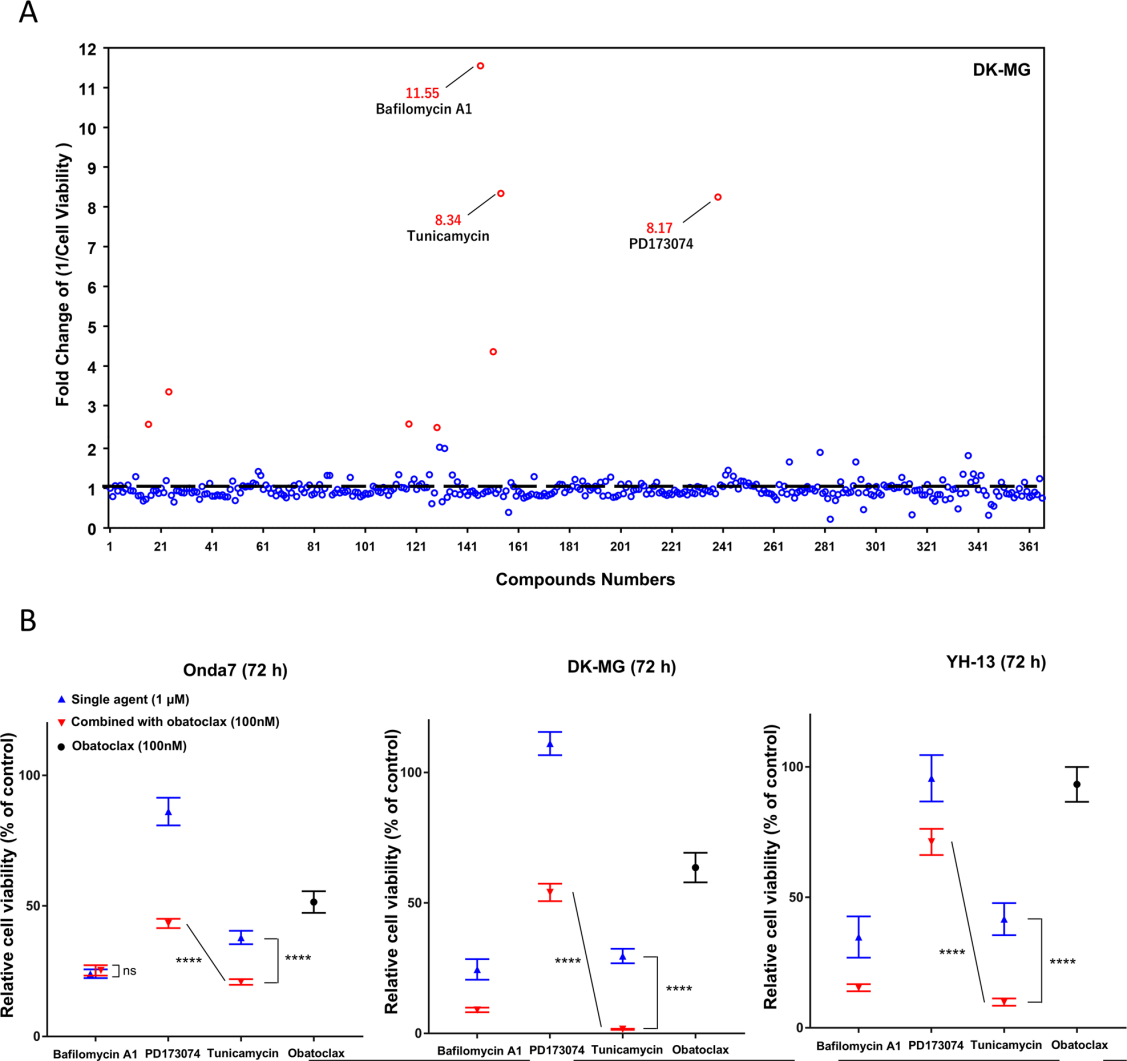
Efficacy of obatoclax was enhanced by potential compounds via inhibitor combination screening. **A** Fold change (1/cell viability) was measured using the CellTiter-Glo assay 72 h after treatment with compounds from the SCADS library with or without obatoclax (200 nM) in the DK-MG cell line. For each data point, the value of (1/cell viability) in the library compounds monotherapy was used to normalize the data. Fold changes larger than 2.0 are marked with red color. **B** Cell viability was measured using the CellTiter-Glo assay 72 h after treatment with 1 μM tunicamycin, bafilomycin A1, or PD173074 with or without 100 nM obatoclax in the Onda7, YH-13, and DK-MG cell lines (*n* = 6). ****P < 0.0001.

Combinations between various Bcl-2 family inhibitors and cytotoxic agents, such as venetoclax combined with cytarabine or hypomethylating agents, have shown significant clinical success in acute myeloid leukemia [44, 45]. Hence, we attempted to identify agents that enhance the inhibitory effects of obatoclax in combination therapy. Accordingly, combination screening was conducted using the SCADS inhibitor library containing 365 compounds with known targets. The viability of DK-MG cells treated with single agents from the SCADS library and in combination with obatoclax was compared and visualized as the fold change in the cell death induction rate (Fig. 3A). Bafilomycin A1, an autophagy inhibitor; Tunicamycin, an ER-stress inducer; and PD173074, an FGFR1 inhibitor, were identified as potential enhancers of obatoclax-induced cytotoxicity. To further investigate their effects on other cell lines, a sensitivity test was conducted with these three candidates and 100 nM of obatoclax (Fig. 3B). Tunicamycin, consistent with its effects on DK-MG cells, also significantly reduced cell viability in Onda7 and YH-13 when combined with obatoclax. In contrast, Bafilomycin A1 failed to induce improvement in obatoclax efficacy in Onda7, whereas PD173074 showed relatively mild inhibition in Onda7 and YH-13. Thus, tunicamycin was identified as a promising candidate for enhancing the inhibitory effects of obatoclax across multiple GBM cell lines.

### Obatoclax synergizes with ER stress inducers to inhibit GBM growth and enhance apoptosis

**Fig. 4:**
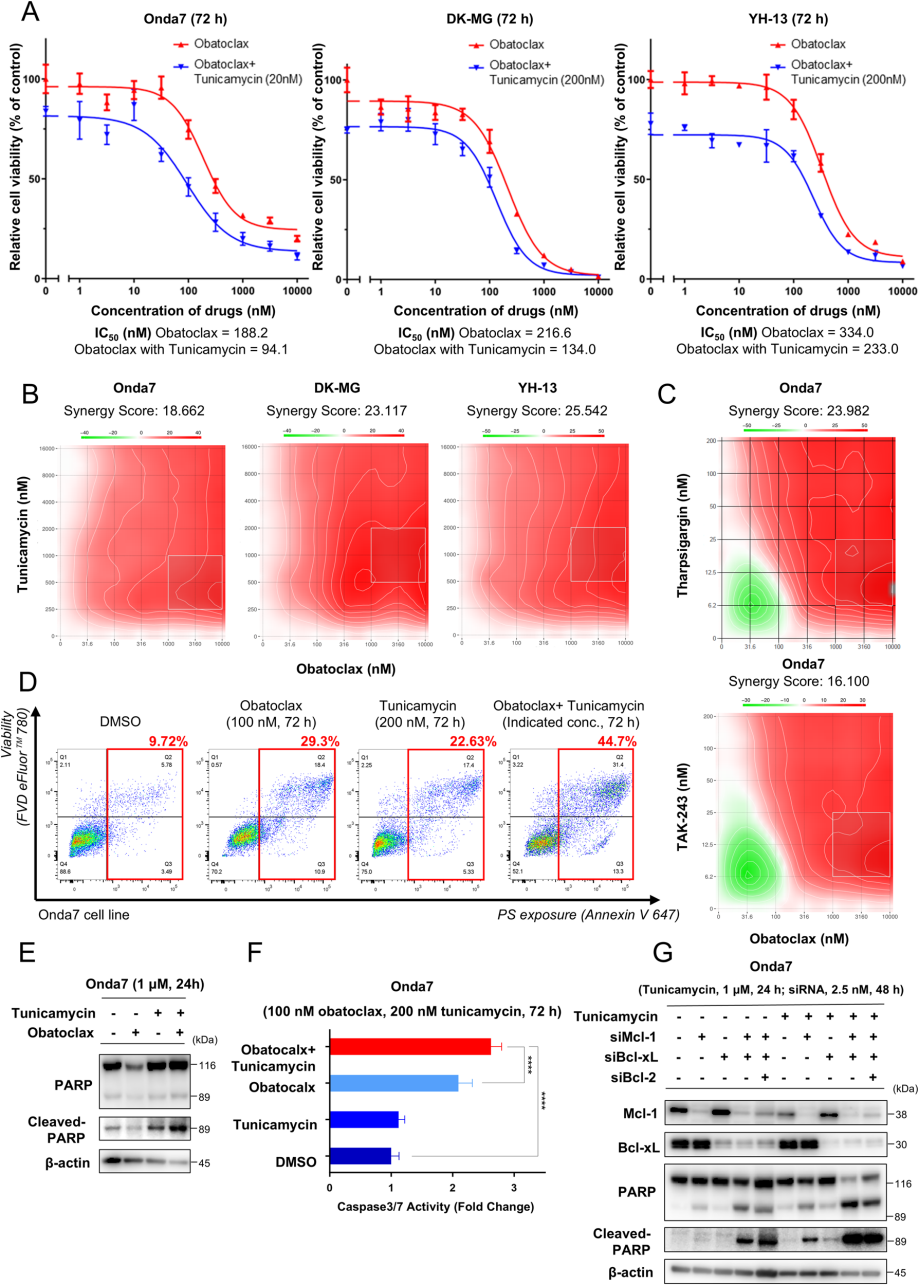
Combination of obatoclax and tunicamycin exerted significant synergistic effects and enhanced apoptosis in GBM cell lines. **A** Comparison of the dose–response curves for obatoclax with or without the indicated concentrations of tunicamycin in the Onda7, DK-MG, and YH-13 cell lines. Cell viability was measured using the CellTiter-Glo assay 72 h after treatment and presented as mean ± SD (*n* = 3). **B** Synergy distribution maps of Onda7, DK-MG, and YH-13 cell lines treated with the indicated concentration of obatoclax and tunicamycin for 72 h. The synergy scores were calculated using the Loewe model. Squares with the white dots indicate areas with the highest Loewe synergy scores. **C** Synergy distribution maps of the Onda7 cell line treated with the indicated concentration of obatoclax and thapsigargin or TAK-243 for 72 h. **D** Evaluation of the cell population under the apoptotic process in the Onda7 cell line treated with a combination of 100 nM obatoclax and 200 nM tunicamycin. Apoptosis was evaluated using Annexin V 647 and FVD eFluor™ 780 staining 72 h after the indicated treatment. The population under the apoptotic process (early stage as Q3, late stage as Q2) are marked with red squares and its percentage is indicated. **E** PARP and its cleavage in Onda7 were detected via western blotting 24 h after treatment with 1 µM obatoclax, 1 µM tunicamycin, or both. **F** The caspase 3/7 activity of Onda7 was measured using the Caspase-Glo 3/7 assay 72 h after the combined treatment with 100 nM obatoclax and 200 nM tunicamycin. Results are presented as mean fold change ± SD (*n* = 8). ****P < 0.0001. **G** Mcl-1, Bcl-xL, PARP, and its cleavage in Onda7 were detected via Western blotting after the indicated treatment with 2.5 nM siNegative, siMcl-1, siBcl-xL, and siBcl-2 with or without 1 µM tunicamycin.

To validate the potential synergy between obatoclax and tunicamycin, single-agent treatments and combination therapy were then compared. The IC_50_ values of both agents were reduced when used together, indicating enhanced efficacy (Fig. 4A and Fig. S3A). Synergy scores offer a quantitative definition for additive effect (−10 < score <10), synergism (score >10), and antagonism (score <−10) [46]. The combination effect, quantified using the Loewe synergy model, confirmed significant synergy between obatoclax and tunicamycin, particularly at various concentrations with high synergy scores (Fig. 4B). Moreover, obatoclax synergized with other ER-stress inducers, including TAK-243 and thapsigargin (Fig. 4C and Fig. S4A). Cell cycle analysis was also performed to analyze proliferation status and cellular response. The results showed that both obatoclax and tunicamycin induced a G1 phase arrest, which was further enhanced under combination treatment (Fig. S5A). Such a G1 blockade prevents cell cycle progression and DNA replication, directly contributing to the reduction in cell proliferation. These results demonstrate that combining obatoclax and tunicamycin effectively inhibits GBM cell growth while consistently showing synergistic cytotoxicity under ER-stress conditions.

Furthermore, combination therapy with obatoclax and tunicamycin enhanced GBM cell apoptosis. The population of apoptotic cells increased after combining treatment with the agents (Fig. 4D and Fig. S4B). Cleavage of PARP increased significantly (Fig. 4E and Fig. S4C) with enhanced caspase 3 and caspase 7 activity (Fig. 4F and Fig. S4D). siRNA knockdown of Mcl-1, Bcl-xL, and Bcl-2 further revealed that cleavage of PARP increased when combined with tunicamycin, particularly with Mcl-1 and Bcl-xL knockdown, suggesting that targeting these genes can synergize with ER-stress induction in GBM (Fig. 4G). These findings demonstrate that the combination of obatoclax and tunicamycin effectively inhibits GBM cell growth and enhances apoptosis, with obatoclax consistently exhibiting synergistic cytotoxicity under ER-stress conditions.

### Combination therapy with obatoclax enhanced ER stress and apoptosis via ATF-4 regulation in GBM

**Fig. 5:**
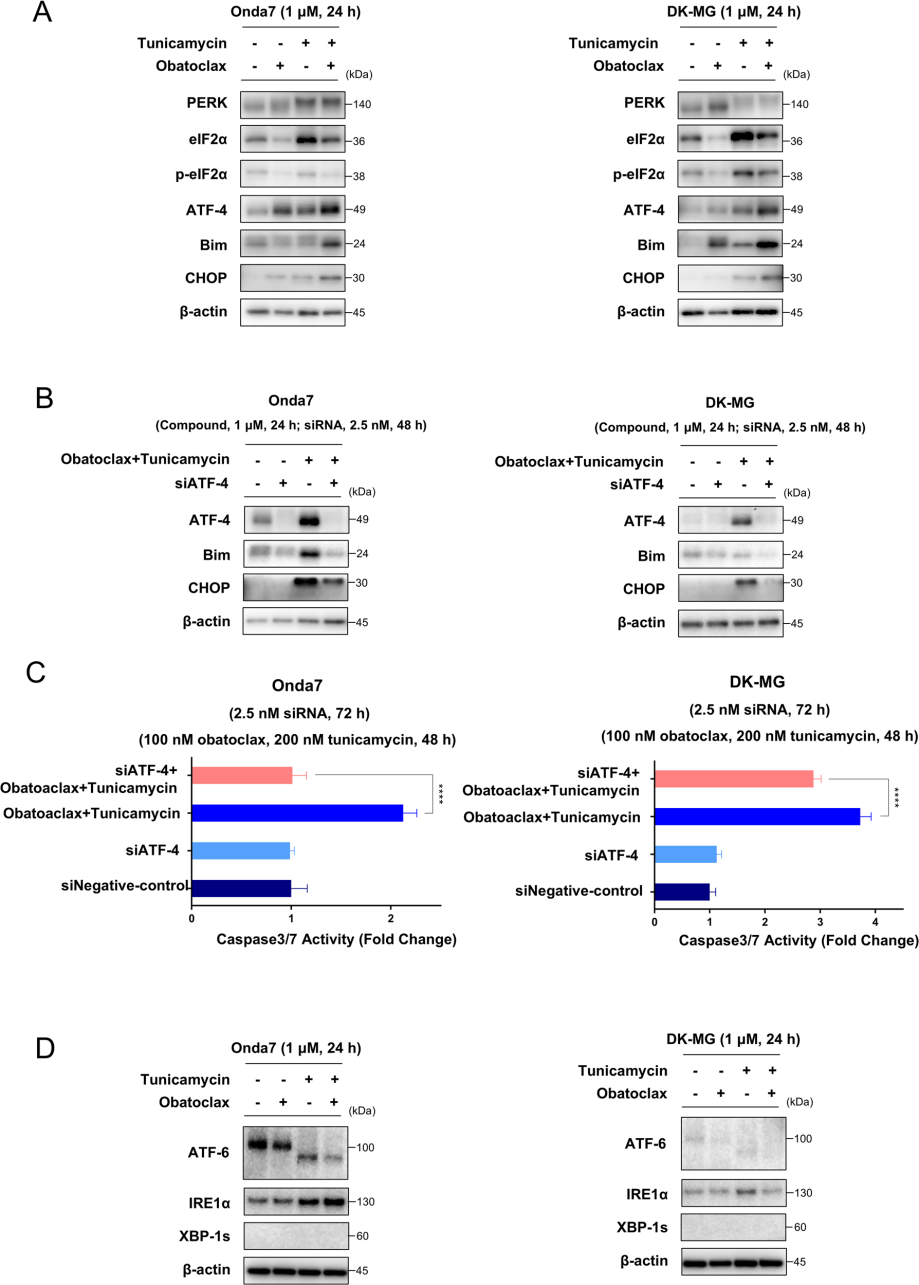
Obatoclax enhanced ER stress and apoptosis via ATF-4-mediated regulation in GBM. **A** PERK pathway-related expression in Onda7 and DK-MG cell lines was detected via Western blotting 24 h after treatment with 1 µM obatoclax, 1 µM tunicamycin, or both. **B** The expression of the ATF-4-Bim-CHOP axis in Onda7 and DK-MG cell lines was detected via Western blotting after indicated treatment with 2.5 nM siATF-4 and combination therapy with 1 µM obatoclax and 1 µM tunicamycin. **C** Caspase 3/7 activity of Onda7 and DK-MG was measured using the Caspase-Glo 3/7 assay after combining treatment with 100 nM obatoclax and 200 nM tunicamycin or treatment with 2.5 nM siATF-4. Results are presented as mean fold change ± SD (*n* = 6). ****P < 0.0001. **D** Additional ER stress pathway-related expression in Onda7 and DK-MG cell lines were detected via Western blotting 24 h after treatment with 1 µM obatoclax, 1 µM tunicamycin, or both.

**Fig. 6:**
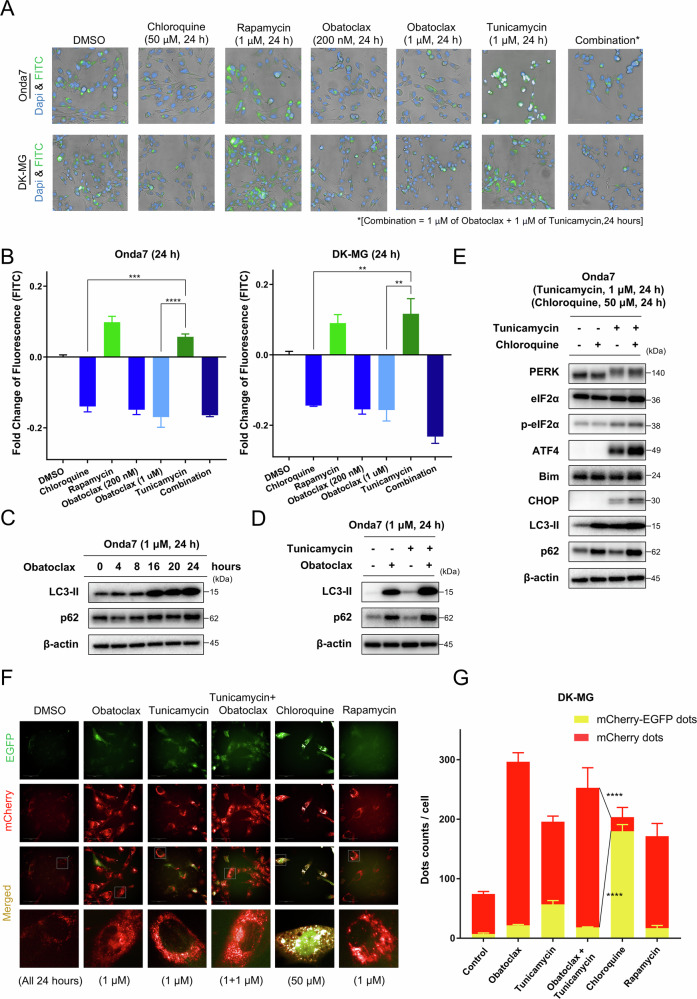
Obatoclax disrupted tunicamycin-induced ER stress via lysosomal dysfunction and enhanced ER stress. **A** Immunofluorescence staining of Onda7 and DK-MG cell lines using Cyto-ID Green (autophagy detector) and Blue-white DPX (nuclear stain) under DAPI and FITC filters. Scale bar: 50 μm. **B** Autophagic flux was calculated as the intensity of fluorescence. Effects on autophagy are presented as fold change of fluorescence. The intensity of fluorescence in the DMSO control group was considered 0% (*n* = 3). **P < 0.01, ***P < 0.001, ****P < 0.0001. **C** LC3-II and p62 expression in the Onda7 cell line was detected via Western blotting after treatment with 1 µM obatoclax for 0, 4, 8, 16, 20, and 24 h. **D** LC3-II and p62 expression in the Onda7 cell line was detected via Western blotting 24 h after treatment with 1 µM obatoclax, 1 µM tunicamycin, or both. **E** PERK pathway-related expression in the Onda7 and DK-MG cell lines were detected via Western blotting 24 h after treatment with 50 µM Chloroquine, 1 µM tunicamycin, or both. **F** Fluorescence microscopy results of the flux in the mCherry-EGFP-LC3 plasmid-transfected DK-MG cell line. After transient transfection with mCherry-EGFP-LC3 plasmids, DK-MG cells were treated with the indicated treatment for 24 h. Scale bar: 100 μm. Concentrations: 1 µM obatoclax, 1 µM tunicamycin, 1 µM rapamycin, 50 µM chloroquine. **G** Quantitative data for yellow (mCherry-EGFP) or red (mCherry) dots in the cells counted (*n* = 4). Dots were detected using the Operetta CLSmachine. ****P < 0.0001.

To clarify the mechanism of action of the combination therapy, the expression levels of ER stress- and UPR pathway-related proteins were measured via Western blotting. For the PERK pathway, shifting of the PERK signal was detected with tunicamycin treatment, indicating the activation of PERK signaling (Fig. 5A). Furthermore, the increased ATF-4 and CHOP expression observed when combining obatoclax and tunicamycin suggested enhanced ER-stress in GBM. The expression of the pro-apoptotic Bcl-2-family protein Bim was also upregulated, alongside CHOP and cleavage of PARP, indicating intense ER stress-induced apoptosis in GBM (Fig. 5A). ATF-4 acts as a regulator of metabolic and redox processes under normal cellular conditions and as a master transcription factor during the stress response. To validate whether ATF-4 upregulation interacted with apoptosis regulation, siRNA-mediated ATF-4 knockdown was performed. Interestingly, ATF-4 knockdown significantly decreased Bim and CHOP expression (Fig. 5B). Decreased caspase-3/7 activity was also observed with ATF-4 knockdown in GBM cells treated with combination therapy, indicating attenuated apoptosis induction (Fig. 5C and Fig. S6A). Moreover, the expression of other ER stress-related pathways, such as ATF-6 and IRE1α, were evaluated. ATF-6 expression decreased whereas IRE1α was relatively upregulated with undetectable expression of downstream XBP-1s, suggesting that ATF-6 and IRE1α pathways were irrelevant to the combination therapy given that the PERK pathway was activated exclusively (Fig. 6D). These results indicate that apoptosis induced by combination therapy with obatoclax and tunicamycin is mediated via an ATF-4-dependent manner accompanied by CHOP/Bim regulation.

### Obatoclax potentially disrupts autophagy in GBM by interrupting cargo degradation

Several reports have indicated that tunicamycin-induced ER stress can activate autophagy to counter cell organ and protein synthesis dysfunctions [47, 48]. We hypothesized that obatoclax can suppress such a protective cellular process to exacerbate ER stress. Cell fluorescent imaging was performed to monitor changes in the autophagic flux using the autophagy detection kit. Monotherapy with obatoclax decreased FITC signals in Onda7 and DK-MG cell lines (Fig. 6A). The impact of obatoclax on fluorescence intensity was highly consistent, suggesting the suppression of the autophagic flux in GBM cells (Fig. 6B). p62 and LC3-II expression were measured via Western blotting. LC3-II accumulation and p62 degradation represent a complete autophagy induction [49]. However, obatoclax treatment promoted the accumulation of both p62 and LC3-II, indicating incomplete autophagy wherein the cargos were not degraded (Fig. 6C and Fig. S7B). These results indicate that obatoclax potentially disrupts autophagy and suppresses the degradation of autophagic cargos, including p62.

### Inhibition of anti-apoptotic Bcl-2 families disrupts the autophagy process, thereby enhancing ER stress with combination therapy

After treatment with 1 μM tunicamycin for 24 h, enhanced autophagic flux was observed, indicating that tunicamycin can induce autophagy in both Onda7 and DK-MG cell lines. However, tunicamycin-induced autophagy was significantly inhibited when cells were treated with obatoclax or combined knockdown of Mcl-1/Bcl-xL/Bcl-2, with decreased FITC signals and fluorescence intensity (Fig. 6A, B and Fig. S7A). Increased p62 and LC3-II accumulation was detected following tunicamycin treatment with either obatoclax or siRNA knockdown, suggesting that inhibition of anti-apoptotic Bcl-2 families disrupted the autophagy process before degradation (Fig. 6D and Fig. S7C, D). Chloroquine, as an autophagy inhibitor, can induce lysosomal dysfunction and prevent autophagic degradation, like obatoclax. Enhanced apoptosis was observed when chloroquine was combined with Mcl-1/Bcl-xL knockdown, indicating that autophagy disruption contributes to the apoptosis induced via anti-apoptotic proteins inhibition (Fig. S7E). After co-treatment with chloroquine and tunicamycin, enhanced ER stress was confirmed via the upregulation of ATF-4 and Bim and CHOP, indicating that autophagy disruption causes severe ER stress and apoptotic gene expression when GBM cells are undergoing ER stress (Fig. 6E). Consistent with these findings, confocal imaging revealed pronounced morphological changes in both mitochondria and ER following co-treatment with obatoclax and tunicamycin, including mitochondrial fragmentation and ER vacuolization, suggesting enhanced ER stress and apoptosis at the organelle level (Fig. S8A).

Furthermore, to further clarify the mechanism underlying autophagy disruption, cells transfected with the mCherry-EGFP-LC3 plasmid were used to monitor the stages of autophagy (Fig. 6F, G). During the autophagy process, the inner acidity of the autolysosome will digest the unstable EGFP-LC3 chain, changing the fluorescent signal from yellow to red. Here, the presence of obatoclax in both monotherapy and combination therapy promoted significantly more counts of mCherry-dots than of mCherry-EGFP-dots, indicating that treated cells had mostly completed their fusion with the lysosome. Thus, the autophagy process was suggested to have been attuned before cargo degradation due to lysosomal dysfunction. These results indicate that inhibition of anti-apoptotic family members causes synergistic cell death by disrupting the protective autophagy process in response to ER stress.

## Discussion

Recognizing the urgent need to improve the therapeutic efficacy of GBM therapies, our current study aimed to uncover novel molecular strategies and their mechanisms. Our in-house inhibitor library allowed us to initially filter promising candidate compounds. The significant difference in efficacy between Bcl-2 family inhibitors obatoclax and navitoclax had attracted our attention. This is consistent with previous studies showing that the co-inhibition of Mcl-1 and other anti-apoptotic proteins, such as Bcl-xL, can significantly induce apoptosis and cell death of melanoma cell lines [50] or sensitize glioma stem cells to other Bcl-2 inhibitors [51].

**Fig. 7:**
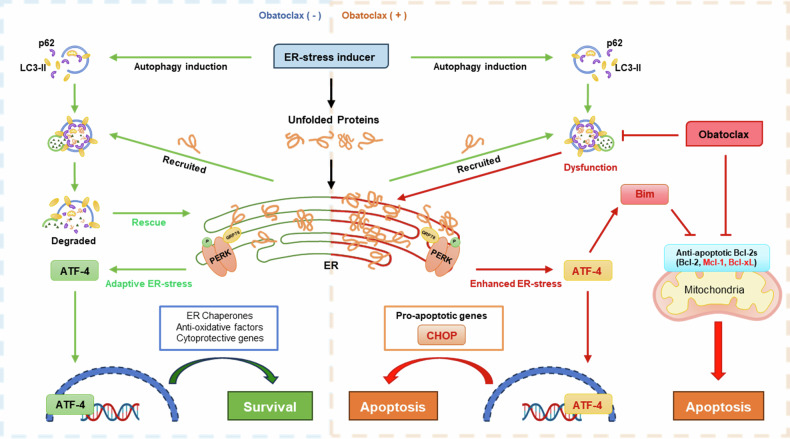
Compact diagram illustrating the summarized mechanism by which obatoclax synergizes with the ER-stress inducer to promote apoptosis in GBM. (Left) Monotherapy with an ER-stress inducer like tunicamycin induces autophagy as a rescue feedback to remove the accumulated unfolded proteins and maintains the stress level in GBM cell lines. Adaptive ER stress regulates the mild expression of ATF-4 via PERK signals, promoting the translation of pro-survival factors under stress conditions. (Right) In the presence of obatoclax, the late stage of autophagy is disrupted via lysosomal dysfunction, causing continuous accumulation of unfolded proteins and enhanced ER stress. The overwhelming stress levels induce significant upregulation of ATF-4, which promotes the translation of pro-apoptotic genes, such as CHOP, and mediates the upregulation of BH3-only protein like Bim. Obatoclax also cooperates with Bim to inhibit anti-apoptotic Bcl-2 families, especially Mcl-1 and Bcl-xL. Such mechanisms contribute to the synergistic apoptosis in GBM.

Reports have shown that combination therapy using inhibitors of anti-apoptotic proteins, such as Bcl-2 family inhibitors, can be a therapeutic strategy for enhancing the efficacy of anticancer treatment in certain hematologic and solid tumors [52, 53]. Our study presents a model in which the inhibition of anti-apoptotic Bcl-2 families synergizes with ER-stress inducers and further promotes apoptosis in different aspects (Fig. 7). Given that ER stress also regulates apoptosis in downstream signaling, the Loewe synergy model was selected for the quantitative assessment of drug interactions based on the additive principle, which gains greater veracity when combinations have similar or identical targets and pathways [54, 55].

UPR is a complex signaling network that restores ER homeostasis by enhancing the protein-folding capacity and reducing the burden of misfolded proteins during ER stress [56]. Studies have also reported that cells under ER-stress conditions tend to induce autophagy to encounter stress in some solid tumors [47, 48]. Recent studies have suggested that autophagy induced via cell stress tends to relieve stress and is therefore more pro-survival and protective of tumor cells and may even contribute to their resistance [34, 57–59]. Processes, like UPR and autophagy, can be commonly recognized as protective mechanisms that help maintain tumor cell survival [60]. However, when stress is prolonged and overwhelming, these pro-survival adaptive responses cause imbalance and induce a lethal process that initiates apoptosis [61]. Although precisely determining the breaking point of this “goldilocks zone” is difficult, inducing ER stress and lethal UPR still remains a potential therapeutic strategy in certain tumors, such as leukemia and GBM [62–65]. Studies have reported that UPR pathways activated alongside severe ER stress activate pro-apoptotic processes under certain conditions [66].

In this model, tunicamycin monotherapy induces the accumulation of unfolded proteins, which can also induce autophagy and result in adaptive ER stress levels. Under such conditions, ATF-4 tended to mediate and promote pro-survival gene translation and helped GBM maintain its homeostasis (Fig. 7, left). However, in the presence of obatoclax, higher ATF-4 and CHOP levels exclusively promoted aggravated ER stress induction, which was intolerable and triggered apoptosis (Fig. 7, right). Evidence suggests that obatoclax can function as a potent late-stage autophagy inhibitor in colorectal cancer [41]. We found that inhibition of Mcl-1/Bcl-xL/Bcl-2 can also disrupt the autophagy process in GBM, causing the accumulation of unfolded proteins and enhancing ER stress. Such overwhelming levels of ER stress had broken the goldilocks point, which triggered the expression of ATF-4-mediated pro-apoptotic genes, such as CHOP and Bim, synergizing with the original function of obatoclax to inhibit anti-apoptotic Bcl-2 families and contributing to the synergistic death of apoptosis (Fig. 7, right).

However, the combination of obatoclax and tunicamycin promoted the accumulation of LC3-II, which commonly indicates autophagy induction. The simultaneous accumulation of p62 certainly helps explain this phenomenon, which indicates that the final degradation of the autophagic cargos was not completed due to disruption by obatoclax. Similarly, enhanced ER stress and pro-apoptotic gene expression were observed. Moreover, a terminated autophagic flux could still be observed when obatoclax monotherapy is applied to GBM cells. Recent studies have suggested that one of the autophagy promoters, beclin-1, directly interacts with anti-apoptotic bcl-2 families, such as bcl-2,bcl-xl, and mcl-1, given that it possesses a similar BH3-like domain [67, 68]. Beclin-1 can be held in an inactive state by forming a complex with mcl-1, while both proteins negatively modulate each other through inverse coregulation [69–71]. The inhibition of mcl-1 releases beclin-1 from its inactive complex into the active autophagy process. However, our results also showed that obatoclax blocks the late stage of autophagy, thus resulting in a terminated autophagic flux. These findings confirmed that inhibition of anti-apoptotic Bcl-2 families disrupts autophagy to avoid ER stress rescue and induce more severe stress conditions, thereby disrupting intracellular homeostasis and contributing to the synergistic effects.

Recent studies involving vivo models have suggested that ER stress promotion and autophagy inhibition can efficiently sensitize GBM to temozolomide therapy and overcome its resistance [72, 73]. Although further investigations into the detailed mechanisms have yet to be conducted, our group is currently planning for such investigations. Furthermore, the mechanisms observed in our model may also extend to other tumor cell types. Although still limited by the potential toxicity toward normal cells in the human body, further application can be achieved by identifying similar and safe chemical compounds.

In conclusion, our study found that obatoclax, as well as the inhibition of anti-apoptotic Bcl-2 families, exhibited synergistic effects with multiple ER stress inducers, which could be a potential therapeutic strategy for GBM treatment. Such a synergistic effect was exerted through autophagy disruption and apoptosis promotion mediated by prolonged ER stress. Our findings underscore potential future possibilities for the treatment of GBM via pro-apoptotic targeting.

## Materials and methods

### Cell lines and cell culture

The Onda7, Onda8, Onda9, NP-2, NP-5 (JCRB, Japan), and DK-MG, 42-MG-BA (Leibniz DSMZ, Germany) cell lines were cultured in DMEM or RPMI 1640 media supplemented with FBS (10–20%), 20 mM HEPES buffer, and antibiotics (kanamycin or penicillin-streptomycin-amphotericin B). The YH-13 (JCRB) cell line was cultured in Minimum Essential Medium Eagle with similar supplements. Cells were maintained at 37 °C under 5% CO_2_.

### Synergy scores and maps

Synergy scores and maps were calculated and generated using the SynergyFinder 3.0 web application [46]. After plating the cells into a 96-well microplate, the desired drug combinations were achieved according to the protocol obtained from the website. The synergistic effects were evaluated via the cell viability assay based on the Loewe reference model. Synergistic scores offer a quantitative definition for additive effect (−10 < score <10), synergism (score >10), and antagonism (score <−10).

Other detailed methods are in the Supplementary Methods and Materials.

## Supplementary information

Supplementary Methods and Materials

Supplementary Figures

Supplementary Table

Uncropped Immunoblot data

## Data Availability

The materials and data obtained in this study will be available upon reasonable request after the completion of a material transfer agreement.
