# Supplementary material for: Inhibition of anti-apoptotic Bcl-2 family members promotes synergistic cell death with ER stress inducers by disrupting autophagy in glioblastoma
Source: Cell Death Discov. 2025 Jul 24;11:340. doi: 10.1038/s41420-025-02632-4 (PMC12289911; doi:10.1038/s41420-025-02632-4)
Supplement: Supplementary file 1 — Supplementary Methods and Materials [file 41420_2025_2632_MOESM1_ESM.docx]

**Supplementary Methods and Materials**

*Cell viability assay*

Cells were seeded into 96-well plates at 2000 cells/well. After 24 h, 40 µL of drug-containing medium was added to achieve the desired concentrations. After a certain period of incubation, 40 µL of CellTiter-Glo® reagent was added, followed by 10 min of agitation and 10 min of equilibration before measuring the luminescence using the TriStar LB 941 plate reader.

*Inhibitor library screening*

GBM cells were seeded into 96-well plates at 2000 cells/well. After 24 h, drugs or DMSO were added at the desired concentrations followed by 72 h of incubation. The sensitivity of each compound was measured using the cell viability assay. Data were analyzed by creating heatmaps using R 4.2.1 and RStudio. Details for each compound are summarized in Supplementary Table 1.

*Western blotting*

Cells were lysed with SDS buffer, heated, vortexed, and centrifuged. Protein quantification was performed using the Pierce BCA Protein Assay Kit (Thermo, 23225). Samples were run on 5%–20% precast gels and transferred at 100 V for 90 min. Membranes were blocked with 5% BSA or Skim Milk/TBST and then incubated with the primary (1:1000) and secondary antibodies (1:10000). Detection was performed using the Amersham Imager 600/800 (GE Healthcare, Japan). The following primary antibodies were used: Mcl-1 (D2W9E), Bcl-xL (54H6), Bcl-2 (D17C4), Bid (2002T), Bim (C34C5), Bax (D2E11), Bak (D4E4), PARP (9642S), Cleaved PARP (D64E10), LC3-II (D11), p62 (5114), PERK (C33E10), p-eIF2α (D9G8), ATF-4 (D4B8), ATF-6 (D4Z8V), IRE1α (14C10), XBP-1s (E9V3E), β-Actin (A1978) (Cell Signaling Technology), eIF2α (Ab5369, Abcam), and CHOP (15204-1-AP, Proteintech).

*siRNA infection*

To achieve a final siRNA concentration of 2.5 nM, 10 µM siRNA was added to 200 µL of Opti-MEM™ (Thermo, 31985-062), followed by 5.0 µL of Lipofectamine™ RNAiMAX (Thermo, 13778-150). After 10 min of incubation at room temperature, the cell suspensions were added. The suspension was then incubated for 3 min before seeding the cells into 96-well plates for viability assays or 60-mm dishes for Western blotting.

siRNA mix targeting MCL1 (L-004501-00-0010), siRNA mix targeting BCL2L1 (L-003458-00-0005), siRNA mix targeting BCL2 (L-003307-00-0005), and siRNA mix targeting ATF-4 (L-005125-00-0005) were used in this study.

*Caspase 3/7 assay*

Cells were seeded into 96-well plates. After 24 h, 40 µL of drug-containing medium was added to achieve the desired concentrations. After a certain period of incubation, 40 µL of Caspase-Glo® 3/7 Assay reagent (PROMEGA, G8091) was added, followed by 10 min of agitation and 60 min of equilibration before measuring luminescence using the TriStar LB 941 plate reader.

*Mitochondrial membrane potential assay*

Cells were seeded into 6-well microplates at a density of 1 × 10^5^ cells/well. After overnight culture, the cells were treated with the indicated drug and siRNA. All floating and adherent cells were collected after the desired duration of drug treatment. The 1 h treatment of 20 μM CCCP (Nacalai, 07253-74) was performed as positive controls for mitochondrial depolarization. Cells were then stained with 200 nM TMRE (Thermo Fischer Scientific, T669) for 30 min at room temperature. Measurement was performed via flow cytometry. FlowJo software (BD Bioscience) was used to analyze and graphically display the data.

*Inhibitors library combination screening*

The Onda7, DK-MG, and YH-13 cell lines were seeded into 96-well microplates, each at a density of 2000 cells/200 µL/well. After incubation for over 24 h, the drugs or DMSO (1 µL) were added to achieve the desired final concentrations. For inhibitor combination, obatoclax was added at a concentration of 100 nM per well into one of every set of parallel groups, whereas the other set served as a parallel control without obatoclax treatment but only library treatment. Following 72 h of incubation, cell survival was evaluated using the cell viability assay.

Screening Committee of Anticancer Drugs (SCADS) inhibitor kit I (version 4.0), II (version 2.1), III (version 1.7), and IV (version 2.4) provided by The Ministry of Education, Culture, Sports, Science and Technology, Japan were used in combination screening. Detailed information on the inhibitor library list can be obtained from http://molpro.jfcr.or.jp/search/library.html

*Annexin V–FVD flow cytometry assay*

Cells were seeded into 6-well microplates at a density of 1 × 10^5^ cells/well. After overnight culture, the cells were treated with the indicated drug concentrations. All floating and adherent cells were collected after the desired duration of drug treatment. Cells were then stained with Fixable Viability Dye eFluor™ 780 and Alexa Fluor 647-conjugated annexin V using an Annexin V/Dead Cell Apoptosis Kit (Thermo Fischer Scientific) for 15 min at room temperature. Measurement was performed via flow cytometry. FlowJo software (BD Bioscience) was used to analyze and graphically display the data.

*Cell cycle analysis*

Cells were seeded into 6-well microplates at a density of 1 × 10^5^ cells/well. After overnight culture, the cells were treated with the indicated drugs. All floating and adherent cells were collected after the desired duration of drug treatment and washed twice with PBS solution. Cells were then stained with Cell Cycle Assay Solution Blue (Dojido, C549) for 30 min at room temperature. Measurement was performed via flow cytometry. FlowJo software (BD Bioscience) was used to analyze and graphically display the data.

*Immunofluorescence*

Cells were cultured on the cover glass in 6-well dishes 72 h before the experiment. The cells on cover glass were carefully treated were treated with the indicated drugs treatment. After 24 h incubation, cells were fixed with 4% paraformaldehyde phosphate buffer solution (Wako) for 15 min, and then permeabilized with 0.1% Triton X-100 in PBS for 10 min, and blocked with 2% BSA in PBS solution for 60 min. The cover glass were then incubated with anti-Mcl-1 Rabbit mAb (D2W9E, #94296, CST, 1:500), anti-Bcl-xL Rabbit mAb (54H6, #2764, CST, 1:500), or Anti-Bcl-2 antibody (ab59348, Abcam, 1:500) with Calnexin Monoclonal antibody (66903-1-Ig, proteintech, 1:500) for overnight incubation, at 4°C. The bound antibody was then incubated with the Alexa 647-conjugated anti-rabbit IgG and Alexa 594-conjugated anti-mouse IgG (Thermo Fisher Science, 1:2000) for 1 h. The samples were then washed with PBS and incubated with Hoechst 33258 (Dojindo, 1 µg/mL) and MitoBright LT Green (Dojindo, 0.1 µM). Images were acquired using the zeiss LSM 700 confocal microscope (Zeiss).

*Autophagic flux assay*

The cells were seeded into 96-well plates at 2000 cells/well and treated with drugs after an overnight culture. The commonly used autophagy inducer rapamycin and the autophagy inhibitor chloroquine were used as the autophagy induction positive and negative controls in the experiments. Autophagy was analyzed using the CYTO-ID® Autophagy Detection Kit 2.0 (Enzo, ENZ-KIT175-0200) according to the manufacturer’s instructions. After staining, the cells were incubated at 37°C for 30 min and then washed and analyzed using the Operetta CLS™ (PerkinElmer, Japan) with FITC and DAPI filters. The autophagic flux was assessed using fluorescence intensity through Harmony® software (Operetta CLS AND Opera Phenix, 4.9).

*mCherry-EGFP-LC3 acidification assessment*

Cells were seeded into 60-mm dishes at 5 × 10^5^ cells/dish, transfected with 2 μg mCherry-EGFP-LC3 plasmids (Bioworld Technology, Minnesota, USA) via FuGENE® HD Transfection Reagent (Promega) for 48 h, and then selected with G418. Cells stably expressing mCherry-EGFP-LC3 were treated with the desired treatment for 24 h and then analyzed using the Operetta CLS™ (PerkinElmer) with mCherry and EGFP filters. The number of stained cells was counted using Harmo8ny® software (Operetta CLS AND Opera Phenix, 4.9).
