# Supplementary material for: Inhibition of anti-apoptotic Bcl-2 family members promotes synergistic cell death with ER stress inducers by disrupting autophagy in glioblastoma
Source: Cell Death Discov. 2025 Jul 24;11:340. doi: 10.1038/s41420-025-02632-4 (PMC12289911; doi:10.1038/s41420-025-02632-4)
Supplement: Supplementary file 2 — Supplementary Figures [file 41420_2025_2632_MOESM2_ESM.pdf]

Supplementary Figure 1

A

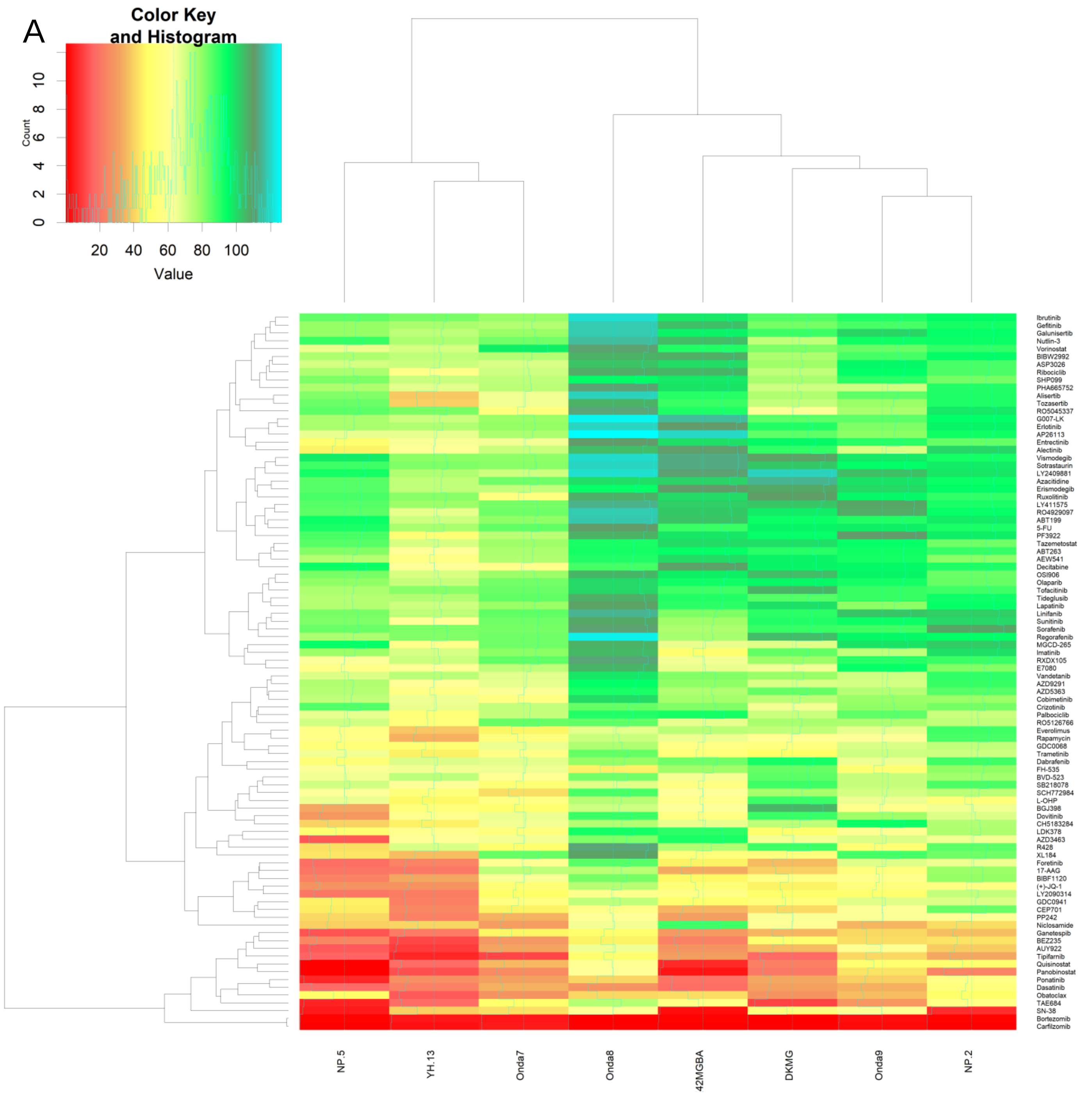

Supplementary Figure S1 Original heatmap of inhibitor library screening.
(A) The original heatmap of inhibitor library screening on 8 GBM cell lines with 92 compounds with known targets. The cell viability in non-treated (DMSO) controls was calculated as 100%. Treatment time was 72 h with concentrations indicated in supplementary table 1.

Supplementary Figure 2

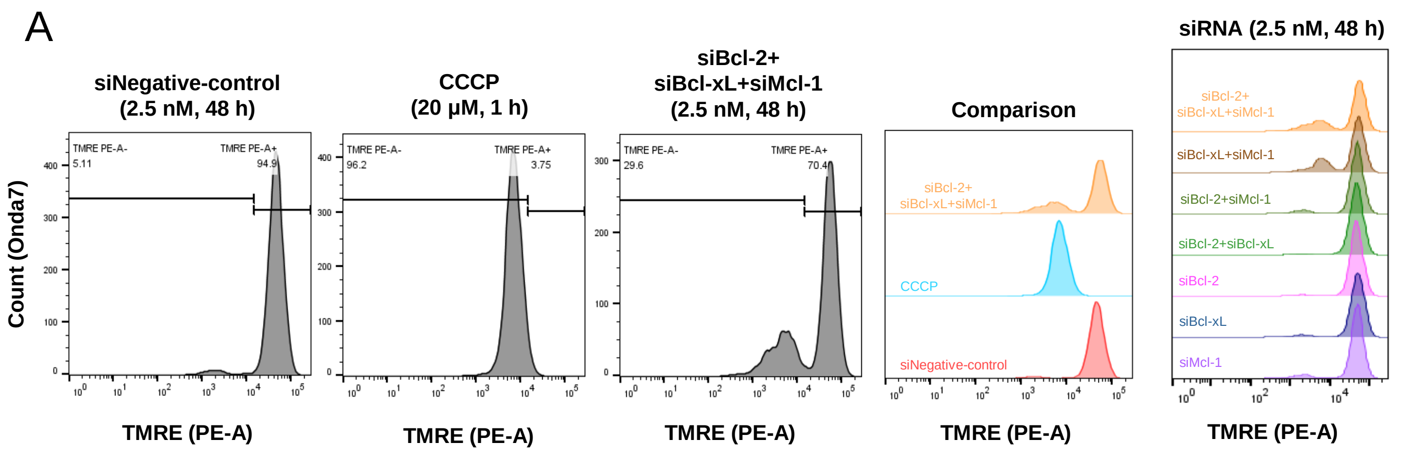

Supplementary Figure S2 Mitochondrial membrane potential changes with combinatorial knockdown of Mcl-1 and Bcl-xL. (A) Mitochondrial membrane potential of Onda7 cells was measured via TMRE fluorescence by flow cytometry after 48 h treatment of 2.5 nM siNegative, siMcl-1, siBcl-xL, or si Bcl-2, or after 1 h treatment of 20 μM CCCP.

Supplementary Figure 3

A

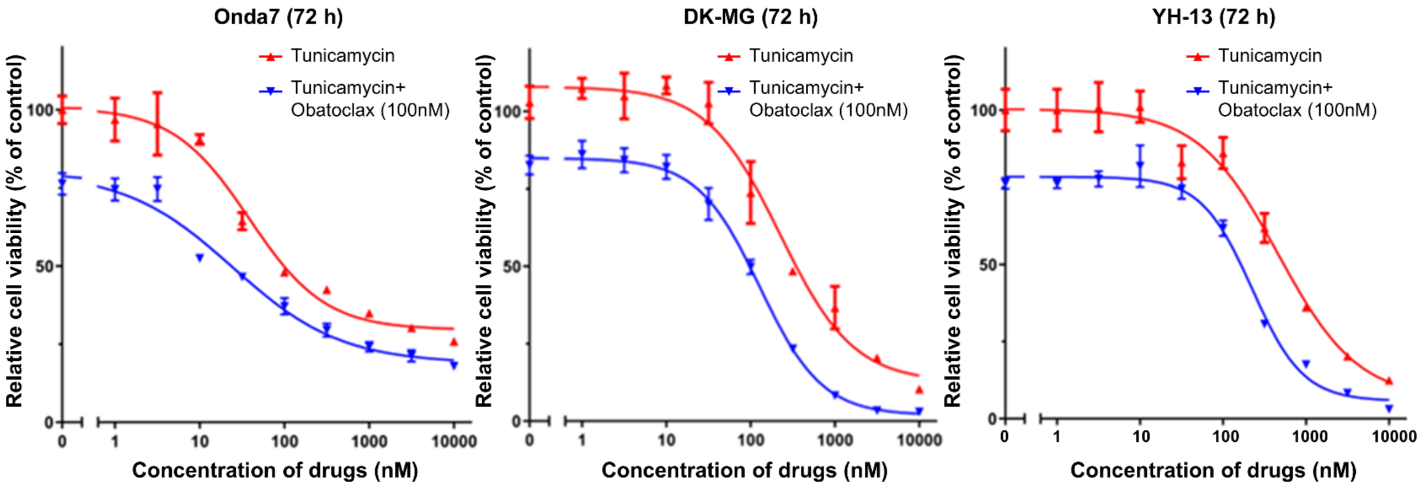

IC_{50} (nM) Tunicamycin = 382.6
Tunicamycin with Obatoclax = 247.3

IC_{50} (nM) Tunicamycin = 223.5
Tunicamycin with Obatoclax = 127.8

IC_{50} (nM) Tunicamycin = 460.5
Tunicamycin with Obatoclax = 223.8

Supplementary Figure S3 Obatoclax reduced the IC_{50} value of tunicamycin in combining treatment.
(A) Comparison of the dose–response curves for tunicamycin in with or without 100 nM obatoclax in Onda7, DK-MG, and YH-13 cell lines. Cell viability was measured using the CellTiter-Glo assay 72 h after treatment and presented as mean ± standard error of the mean (SD) (n = 3).

Supplementary Figure 4

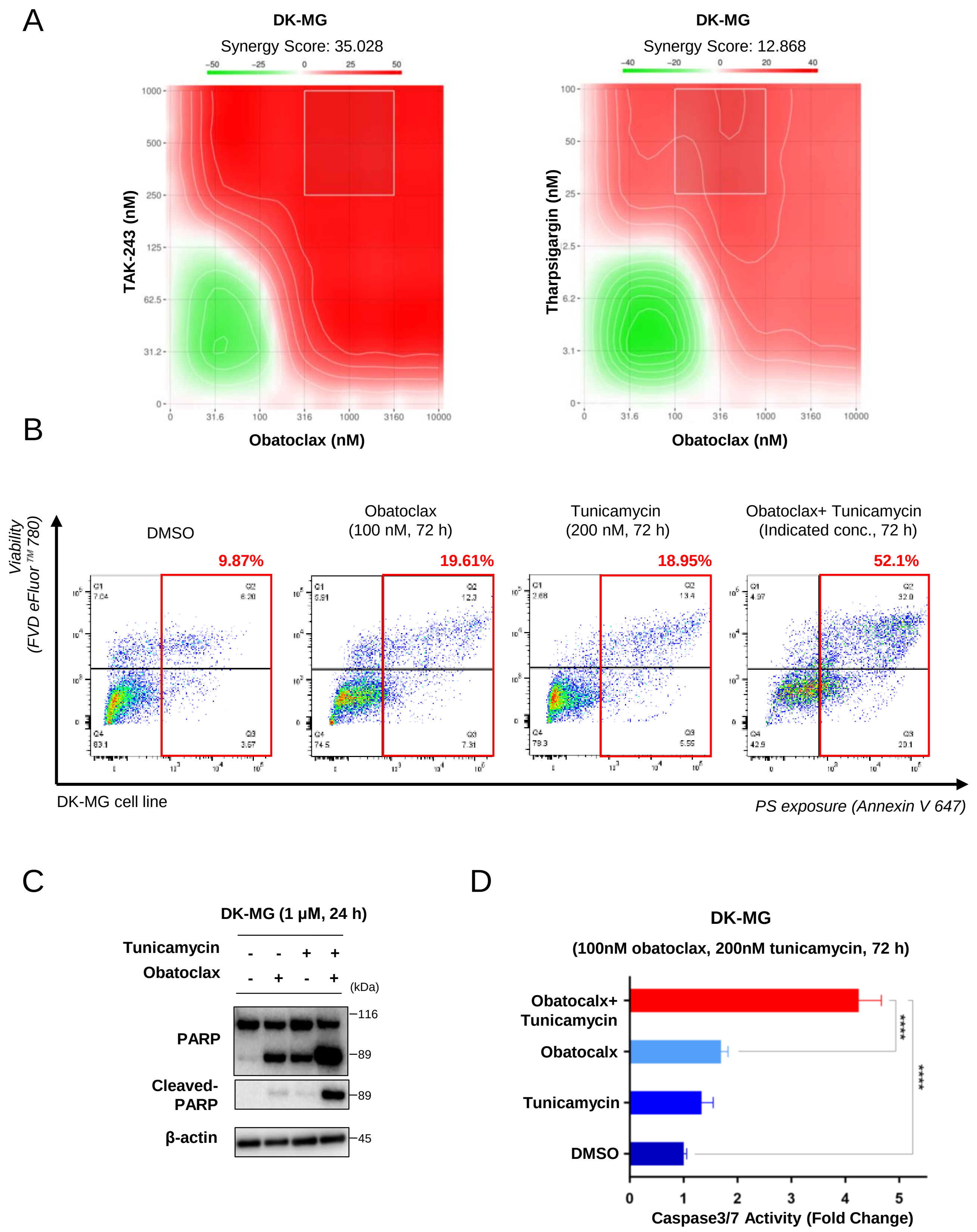

Supplementary Figure S4 Combination of obatoclax and tunicamycin exerted significant synergistic effects and enhanced apoptosis in GBM cell lines.

(A) Synergy distribution maps of the Onda7 cell line treated with the indicated concentration of obatoclax and thapsigargin or TAK-243 for 72 h. (B) Evaluation of the cell population under the apoptotic process of the DK-MG cell line treated with a combination of 1 µM obatoclax and 1 µM tunicamycin. Apoptosis was evaluated using Annexin-V and FVD staining after 72 h of the indicated treatment. (C) PARP and its cleavage in DK-MG were detected via Western blotting 24 h after treatment of 1 µM obatoclax, 1 µM tunicamycin, or both. (D) Caspase 3/7 activity of DK-MG was measured using the Caspase-Glo 3/7 assay 72 h after treatment with a combination of 1 µM obatoclax and 1 µM tunicamycin. Results are presented as mean fold change ± standard error of the mean (SD) (n = 8). ****P < 0.0001.

Supplementary Figure 5

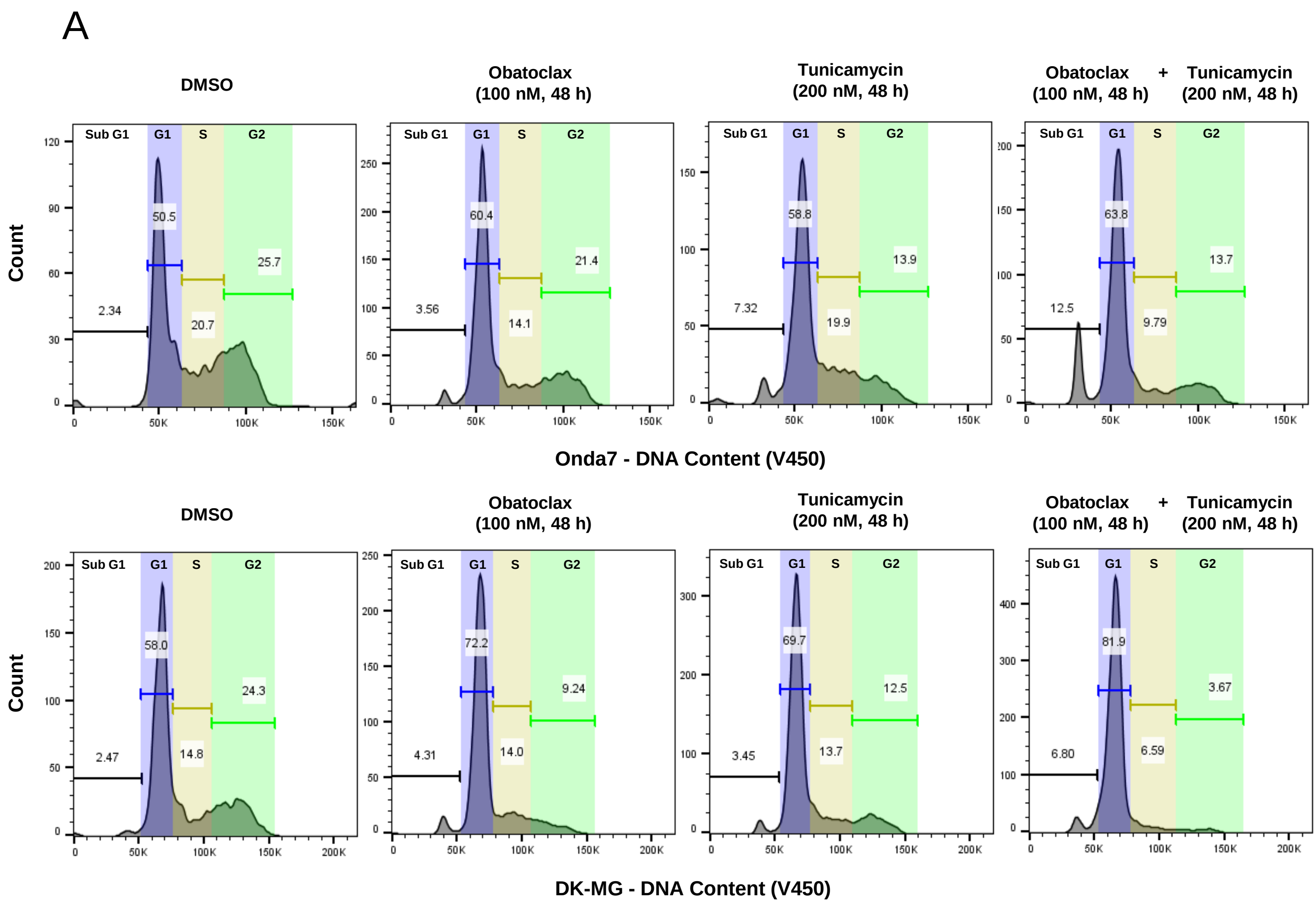

Supplementary Figure S5 Combination therapy of obatoclax and tunicamycin induced enhanced G1 phase arrest in GBM. (A) Cell cycle analysis by flow cytometry for Onda7 and DK-MG cell lines after 48 h treatment with 100 nM obatoclax, 200 nM tunicamycin, or both. Distributions of cells in sub-G1, G1, S, G2 phases were marked with numbers of percentages.

Supplementary Figure 6

A

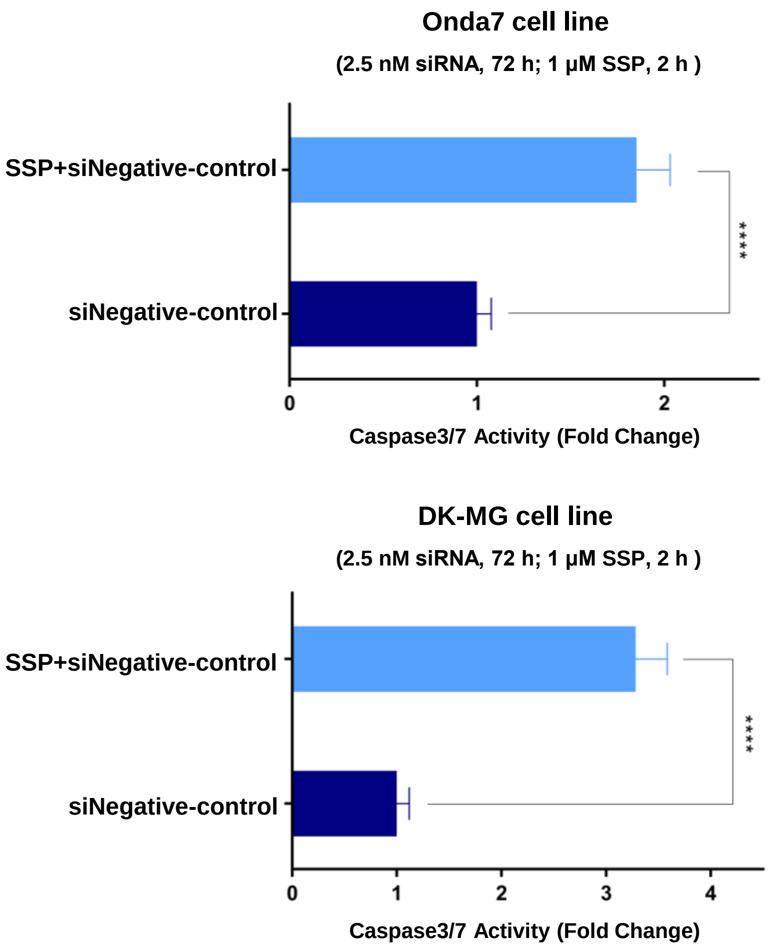

Supplementary Figure S6 Positive control for apoptosis induction in siRNA treatment.
(A) Caspase 3/7 activity of Onda7 and DK-MG was measured using the Caspase-Glo 3/7 assay after 72 h treatment of 2.5 nM siATF-4 with or without 2 h treatment of 1 µM Staurosporine (SSP) as positive control for apoptosis induction. Results are presented as mean fold change ± standard error of the mean (SD) (n = 6). ****P < 0.0001.

Supplementary Figure 7

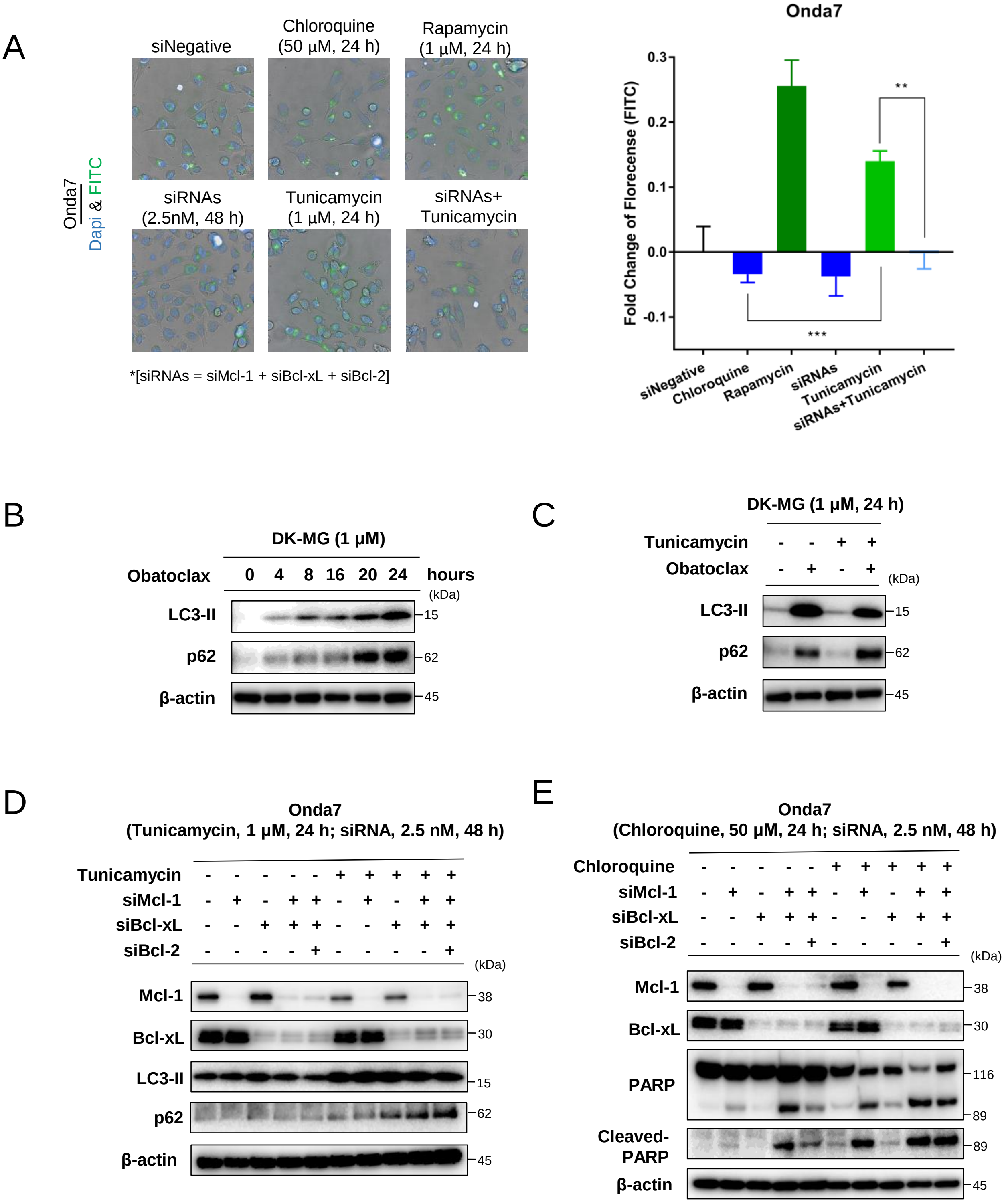

Supplementary Figure S7 Knockdowns of Mcl-1 and Bcl-xL disrupted tunicamycin-induced autophagy via lysosomal dysfunction and enhanced ER stress.

(A) Immunofluorescence staining of Onda7 cell line using Cyto-ID Green (autophagy detector) and Blue-white DPX (nuclear stain) under DAPI and FITC filters. Scale bar: 50 µm. Autophagic flux was calculated as the intensity of FITC fluorescence. Effects on autophagy are presented as fold change of fluorescence. The intensity of FITC fluorescence in the control group was considered 0% (n = 3). **$P < 0.01$, ***$P < 0.001$. (B) LC3-II and p62 expression in the DK-MG cell line was detected via western blotting after treatment with 1 µM obatoclax for 0, 4, 8, 16, 20, and 24 h. (C) LC3-II and p62 expression in the DK-MG cell line were detected using western blotting 24 h after treatment with 1 µM obatoclax, 1 µM tunicamycin, or both. (D) LC3-II and p62 expression in Onda7 was detected via western blotting after the indicated treatment with siNegative, siMcl-1, siBcl-xL, and siBcl-2 with or without 1 µM tunicamycin. (E) PARP and its cleavage in Onda7 was detected via western blotting after the indicated treatment with siNegative, siMcl-1, siBcl-xL, and siBcl-2 with or without 50 µM chloroquine.

Supplementary Figure 8

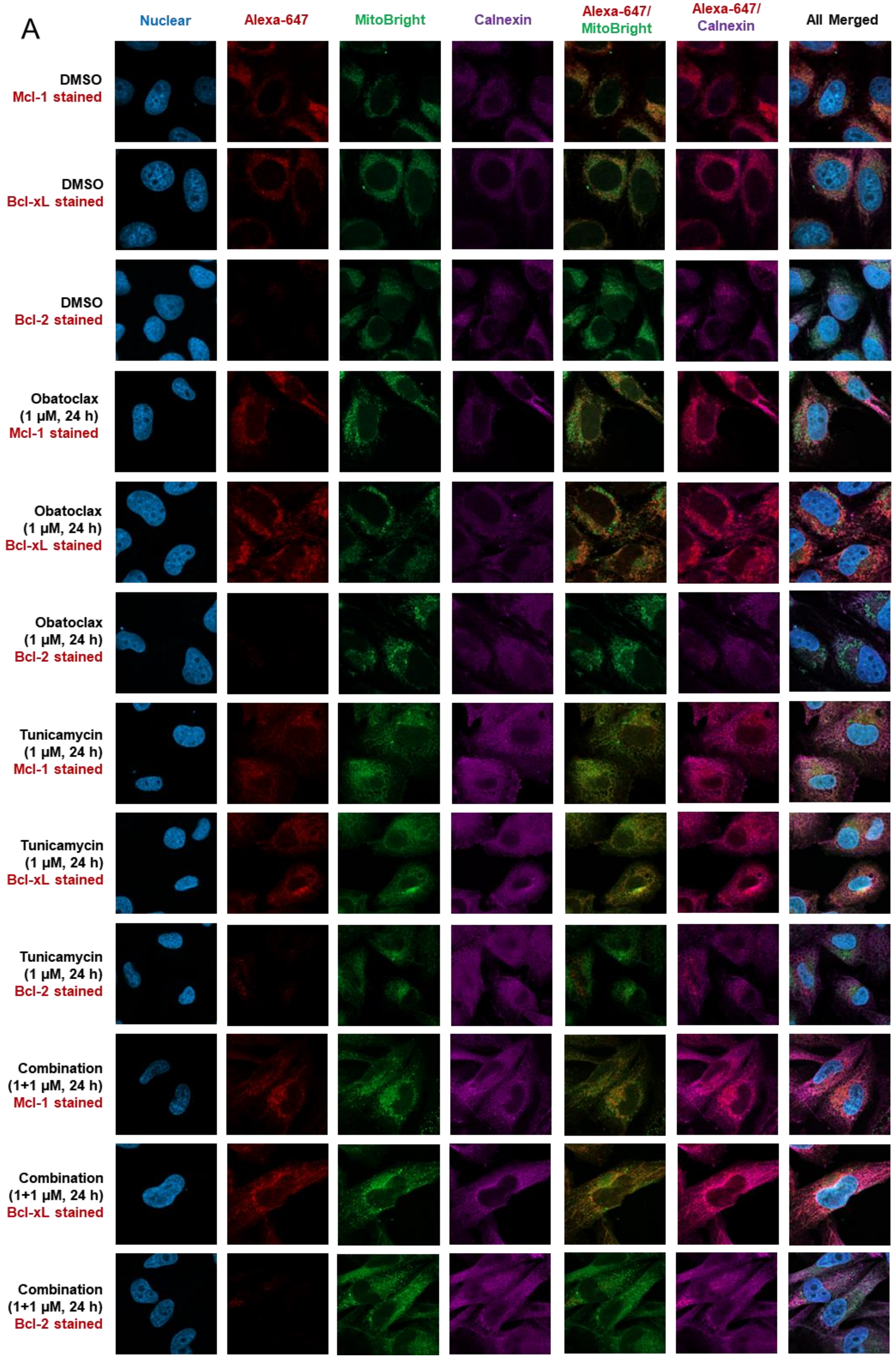

Supplementary Figure S8 Immunofluorescence imaging of Mcl-1, Bcl-xL, Bcl-2, with mitochondria and ER structure under the treatment.
(A) Representation of confocal microscopy images of Onda7 cell lines after 24 h treatment with DMSO, or 1 μM obatoclax, 1 μM tunicamycin, or both. Immunofluorescence staining was performed by using filters of Dapi (Hoechst 33342 - nuclear staining), Alexa-647 (Alexa-647 - Mcl-1, Bcl-xL, Bcl-2 staining), EGFP (MitoBright Green - Mitochondria staining), and Cy5 (Alexa-594 - Calnexin staining for ER structure).
