# Supplementary material for: Inhibition of anti-apoptotic Bcl-2 family members promotes synergistic cell death with ER stress inducers by disrupting autophagy in glioblastoma
Source: Cell Death Discov. 2025 Jul 24;11:340. doi: 10.1038/s41420-025-02632-4 (PMC12289911; doi:10.1038/s41420-025-02632-4)
Supplement: Supplementary file 3 — Supplementary Table [file 41420_2025_2632_MOESM3_ESM.docx]

| **Inhibitors** | **Main targets** | **Final concentration** | **Company** |
| --- | --- | --- | --- |
| Ganetespib | HSP90 | 0.1 µM | Adooq Bioscience |
| Dabrafenib | BRAF/CRAF | 1 µM | Adooq Bioscience |
| BEZ235 | PI3K/mTOR | 1 µM | Adooq Bioscience |
| RO5126766 | RAF/MEK | 1 µM | Adooq Bioscience |
| Cobimetinib | MEK | 0.1 µM | Adooq Bioscience |
| Trametinib | MEK | 0.1 µM | Adooq Bioscience |
| SCH772984 | ERK | 1 µM | Adooq Bioscience |
| BVD-523 | ERK | 1 µM | Adooq Bioscience |
| GDC0068 | AKT | 1 µM | Adooq Bioscience |
| Navitoclax (ABT263) | Bcl-xL/Bcl2 | 1 µM | Adooq Bioscience |
| Obatoclax | Bcl-xL/Bcl2/Mcl1 | 1 µM | Adooq Bioscience |
| Venetoclax (ABT199) | Bcl-xL/Bcl2 | 1 µM | Adooq Bioscience |
| Decitabine | DNA Methyltransferase | 1 µM | Adooq Bioscience |
| Azacitidine | DNA Methyltransferase | 1 µM | Adooq Bioscience |
| Vorinostat | HDAC | 1 µM | Adooq Bioscience |
| Panobinostat | HDAC | 0.1 µM | Adooq Bioscience |
| Quisinostat | HDAC | 0.1 µM | ShangHai Biochempartner |
| Tazemetostat | EZH2 | 1 µM | Adooq Bioscience |
| (+)-JQ-1 | BET | 1 µM | ShangHai Biochempartner |
| Sotrastaurin | panPKC | 1 µM | Adooq Bioscience |
| Nutlin-3 | MDM2 | 1 µM | Adooq Bioscience |
| RO5045337 | MDM2 | 1 µM | Adooq Bioscience |
| Ruxolitinib | JAK | 1 µM | Adooq Bioscience |
| Tofacitinib | JAK | 1 µM | Adooq Bioscience |
| Palbociclib | CDK4/6 | 1 µM | Adooq Bioscience |
| Ribociclib | CDK4/6 | 1 µM | Adooq Bioscience |
| Alisertib | aurora | 1 µM | Adooq Bioscience |
| Tozasertib | Panaurora | 1 µM | Adooq Bioscience |
| RO4929097 | Γsecretase | 1 µM | Adooq Bioscience |
| LY411575 | Γsecretase | 1 µM | Adooq Bioscience |
| LY2090314 | GSK3ab | 1 µM | Adooq Bioscience |
| Tideglusib | GSK3b | 1 µM | Adooq Bioscience |
| Olaparib | PARP | 1 µM | ShangHai Biochempartner |
| Ibrutinib | Btk | 1 µM | Adooq Bioscience |
| Erismodegib | Smo | 1 µM | ShangHai Biochempartner |
| Vismodegib | Smo | 1 µM | Adooq Bioscience |
| Bortezomib | proteasome | 0.1 µM | Adooq Bioscience |
| Carfilzomib | proteasome | 0.1 µM | Adooq Bioscience |
| Niclosamide | STAT3 | 1 µM | ShangHai Biochempartner |
| OSI906 | IGF1R | 1 µM | Adooq Bioscience |
| 5-FU | DNA/RNA synthesis | 100 µM | Adooq Bioscience |
| SN-38 | TOPO1 | 0.5 µM | Adooq Bioscience |
| SHP099 | SHP2 | 5 µM | ShangHai Biochempartner |
| Regorafenib | VEGFR | 1 µM | Adooq Bioscience |
| G007-LK | Tankyrase | 2 mM | ShangHai Biochempartner |
| LY2409881 | IKK | 1 µM | Adooq Bioscience |
| Entrectinib | Trk/ROS1/ALK | 1 µM | Adooq Bioscience |
| Dovitinib | FLT3 | 1 µM | Adooq Bioscience |
| MGCD-265 | Tie2 | 1 µM | Adooq Bioscience |
| Galunisertib | TGFβ | 1 µM | Adooq Bioscience |
| Linifanib | KDR/CSF-1R/ | 1 µM | Adooq Bioscience |
|  | FLT-1/3 |  |  |
| AZD3463 | ALK/IGF1R | 1 µM | Bio Vision |
| AZD5363 | AKT | 1 µM | Adooq Bioscience |
| AUY922 | HSP90 | 0.1 µM | ShangHai Biochempartner |
| R428 | Axl | 1 µM | ShangHai Biochempartner |
| RXDX105 | RET/BRAF | 1 µM | ShangHai Biochempartner |
| Crizotinib | ALK/MET | 1 µM | ShangHai Biochempartner |
| Ceritinib (LDK378) | ALK | 1 µM | ActiveBiochem |
| Alectinib | ALK | 1 µM | ActiveBiochem |
| TAE684 | ALK | 1 µM | ChemieTek |
| AP26113 | ALK | 1 µM | ShangHai Biochempartner |
| Lorlatinib (PF3922) | ALK/ROS1 | 1 µM | ActiveBiochem |
| ASP3026 | ALK | 1 µM | ChemieTek |
| XL184 | VEGFR2/MET/RET | 1 µM | ActiveBiochem |
| Vandetanib | EGFR/VEGFR | 1 µM | ShangHai Biochempartner |
| E7080 | VEGFR2 | 1 µM | Selleck |
| CEP701 | FLT3/JAK2 | 1 µM | Calbiochem |
| Foretinib | VEGFR2/MET/ | 1 µM | Adooq Bioscience |
|  | ROS1 |  |  |
| Afatinib (BIBW2992) | EGFR | 0.1 µM | ChemieTek |
| Erlotinib | EGFR | 1 µM | LC laboratories |
| Gefitinib | EGFR | 1 µM | LC laboratories |
| Lapatinib | EGFR/HER2 | 2 µM | LC laboratories |
| Osmertinib (AZD9291) | EGFR | 1 µM | Selleck |
| PHA665752 | MET | 1 µM | Tocris Bioscience |
| AEW541 | IGF1R | 1 µM | ActiveBiochem |
| Sorafenib | VEGFR2/RAF | 1 µM | Selleck |
| Sunitinib | VEGFR2/PDGFR | 1 µM | Selleck |
| BIBF1120 | VEGFR2/FGFR | 1 µM | Selleck |
| CH5183284 | FGFR | 1 µM | ActiveBiochem |
| BGJ398 | FGFR | 1 µM | ShangHai Biochempartner |
| Ponatinib | bcr-abl/Src/FGFR | 1 µM | Selleck |
| Imatinib | bcr-abl/KIT | 1 µM | LC laboratories |
| 17-AAG | HSP90 | 1 µM | LC laboratories |
| GDC0941 | PI3K | 1 µM | LC laboratories |
| Rapamycin | mTOR | 1 µM | AG Scientific |
| Everolimus | mTOR | 1 µM | Chem Scene |
| PP242 | mTOR | 1 µM | Adooq Bioscience |
| SB218078 | CHK1 | 1 µM | Tocris Bioscience |
| Dasatinib | bcr-abl/Src | 1 µM | Selleck |
| FH-535 | Wnt/TCF | 1 µM | Adooq Bioscience |
| Tipifarnib | FTPase | 1 µM | Adooq Bioscience |
| L-OHP | DNA synthesis | 1 µM | wako |

**Supplementary Table 1 Information List of the Inhibitor Library**

Information of each inhibitor and their main targets, screening concentrations, original companies are included in this list. The solvent for each inhibitor was dimethyl sulfoxide (DMSO), with sterile water being used for palbociclib only.
