# Supplementary material for: Inhibition of anti-apoptotic Bcl-2 family members promotes synergistic cell death with ER stress inducers by disrupting autophagy in glioblastoma
Source: Cell Death Discov. 2025 Jul 24;11:340. doi: 10.1038/s41420-025-02632-4 (PMC12289911; doi:10.1038/s41420-025-02632-4)

Figure 2A
Original Data

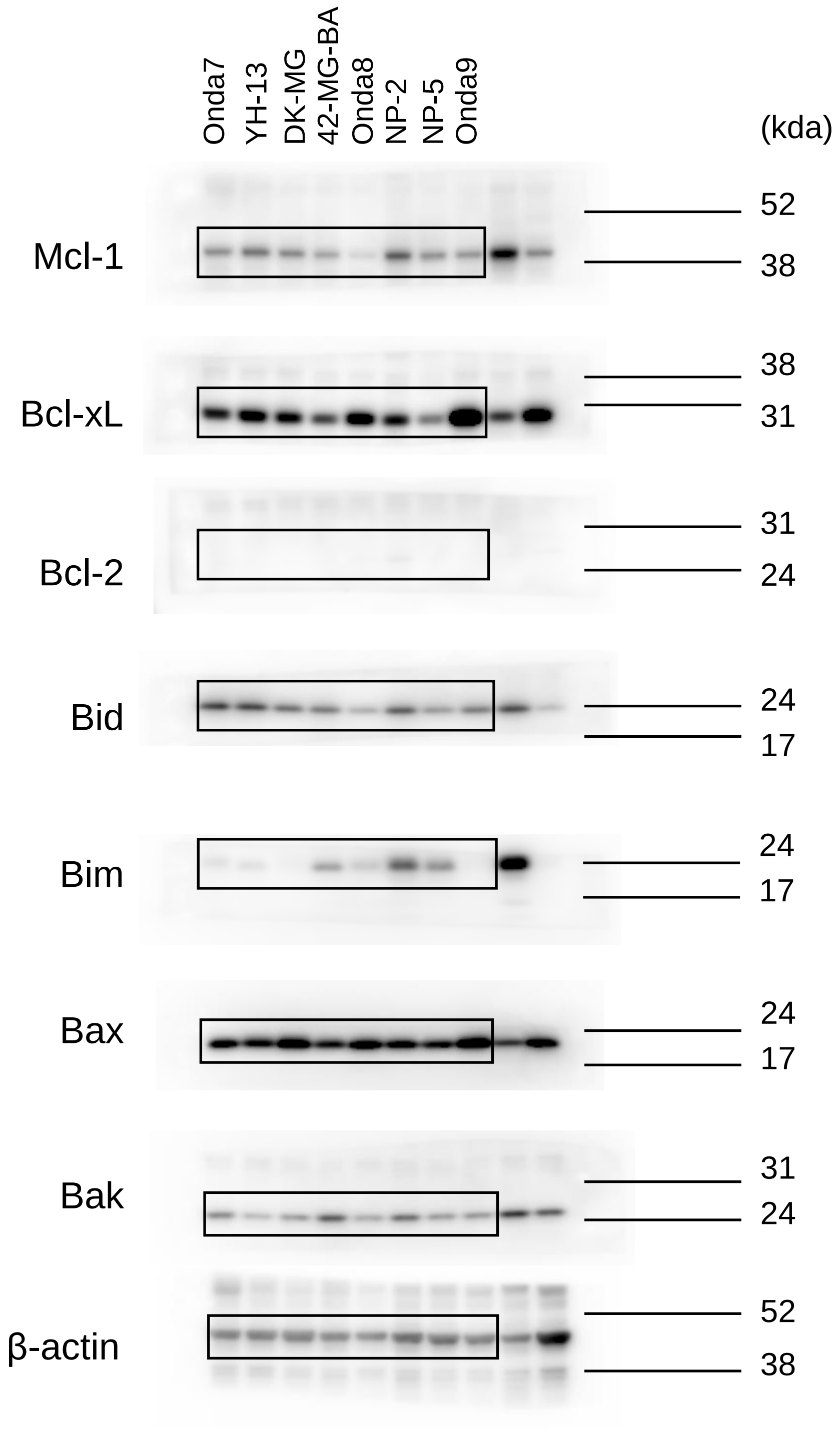

Figure 2B-1
Original Data

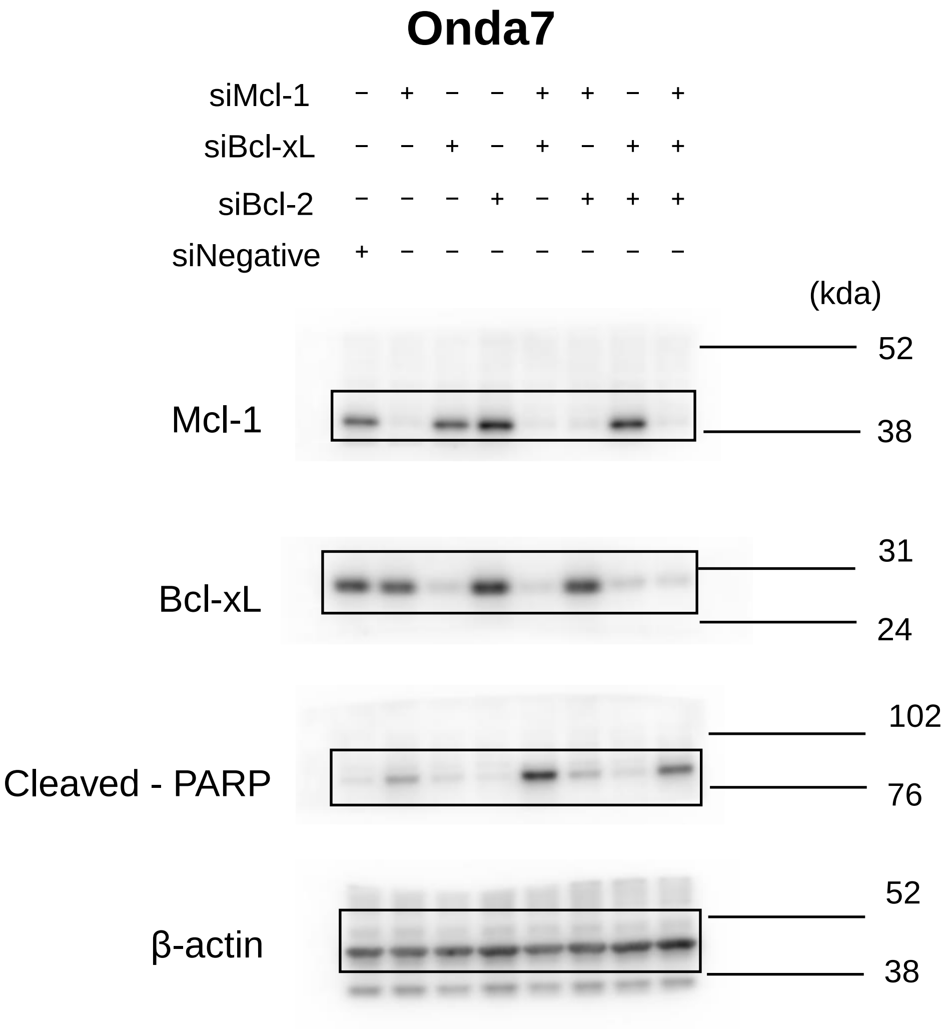

Figure 2B-2
Original Data

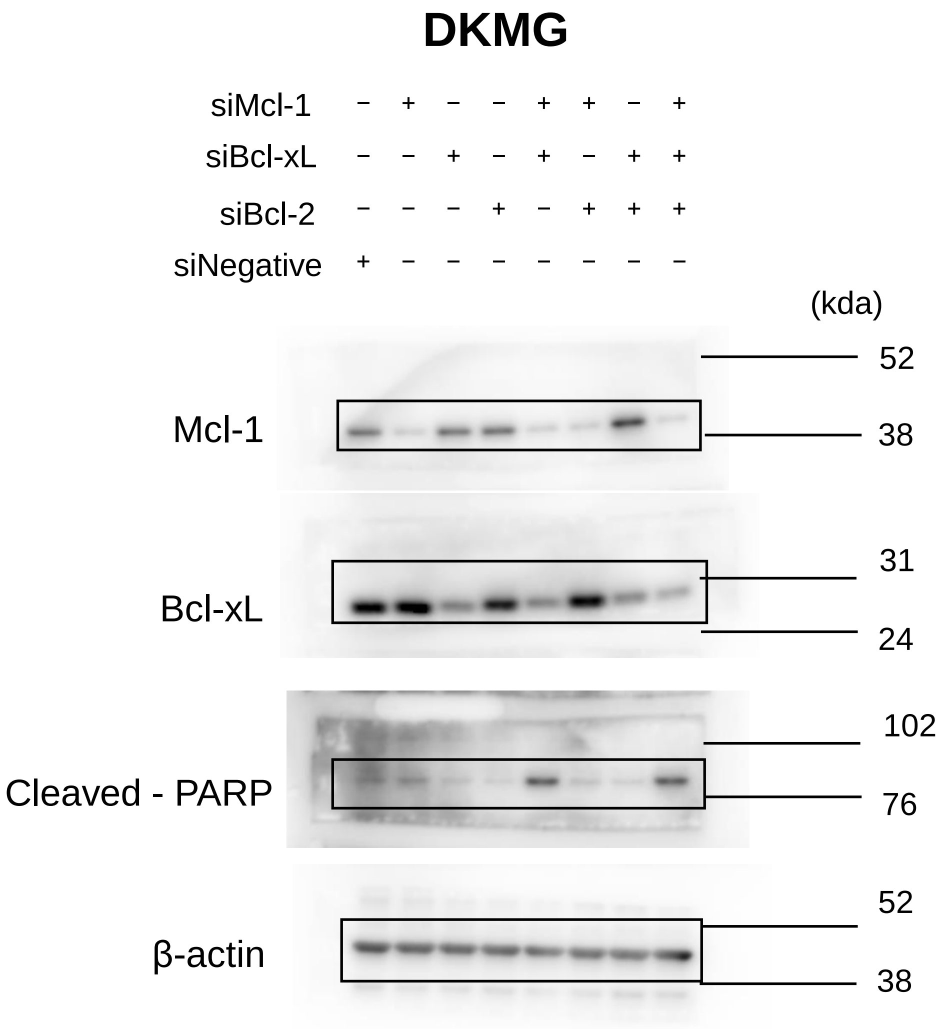

Figure 4E
Original Data

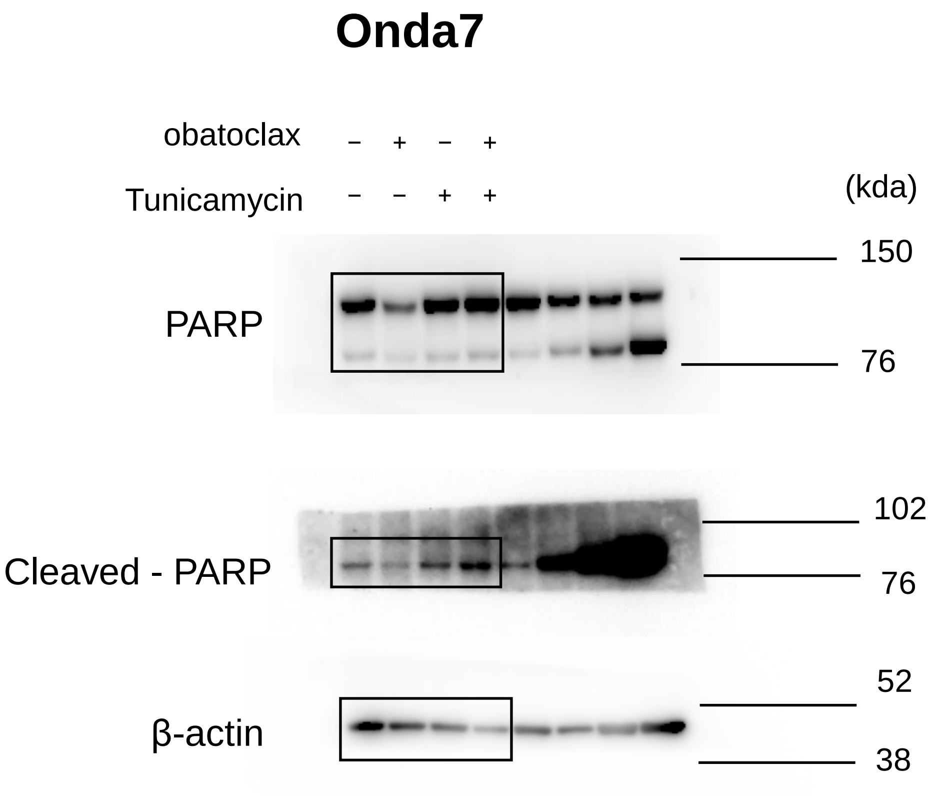

Figure 4G
Original Data

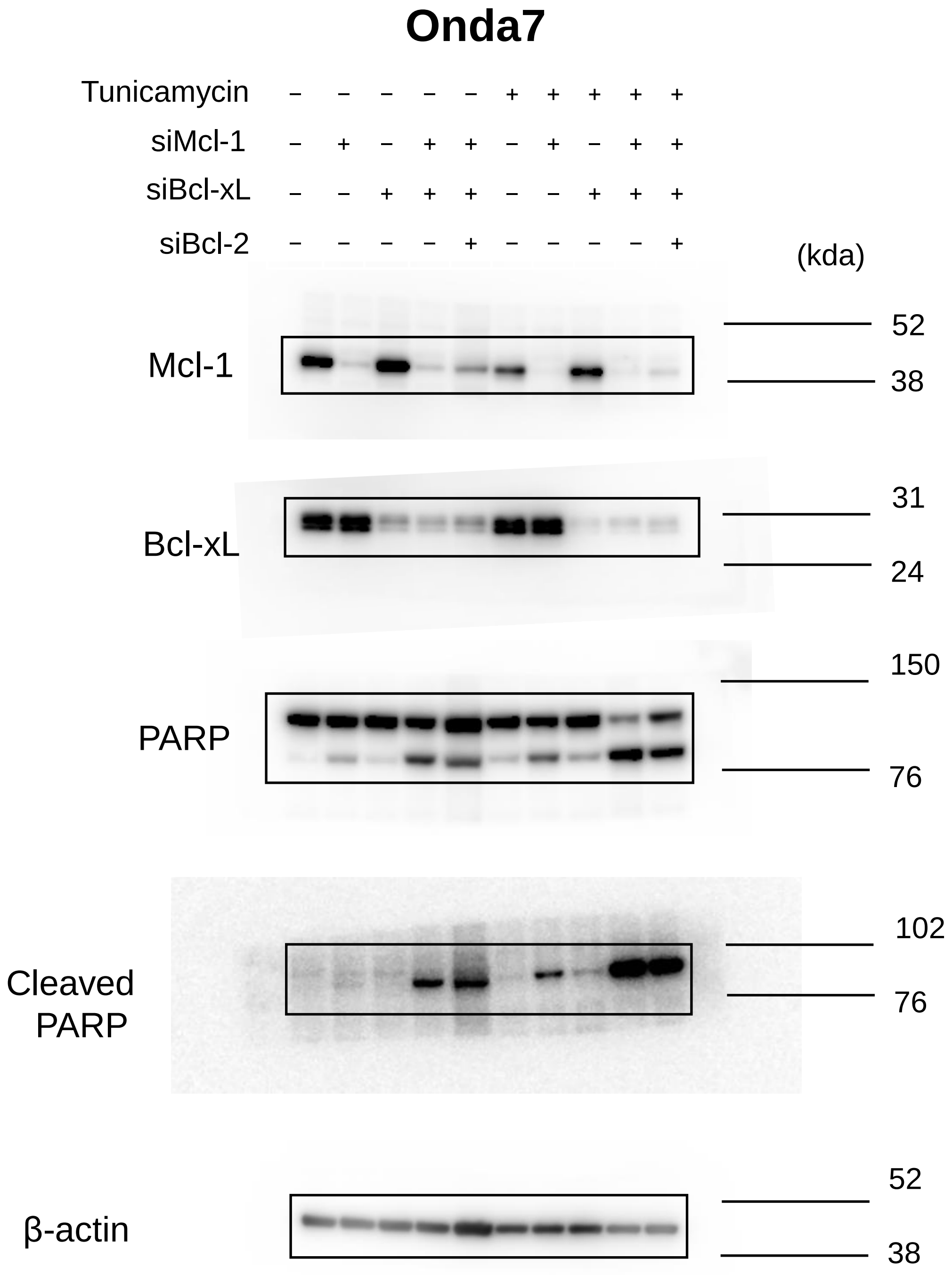

Figure 5A-1
Original Data

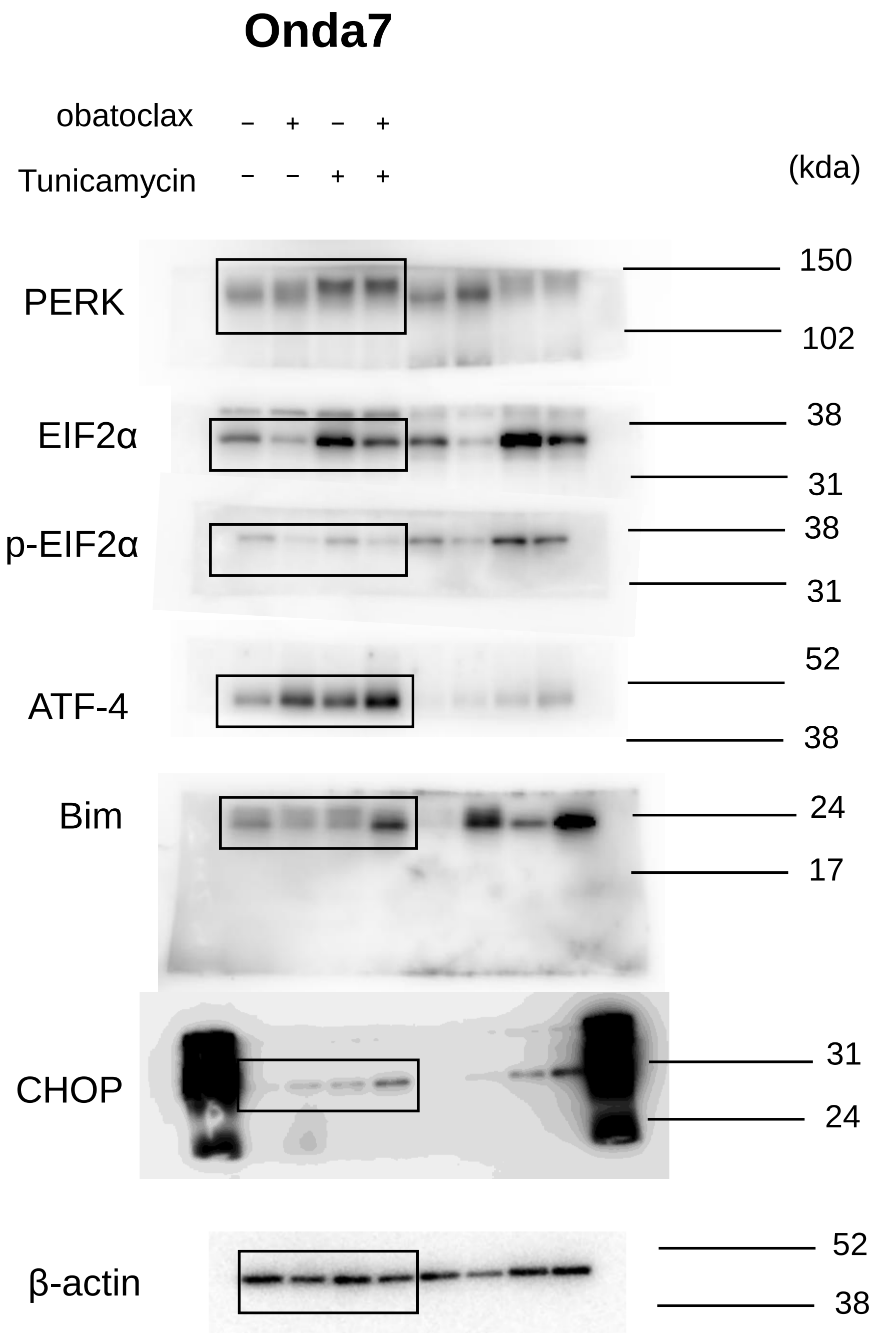

Figure 5A-2
Original Data

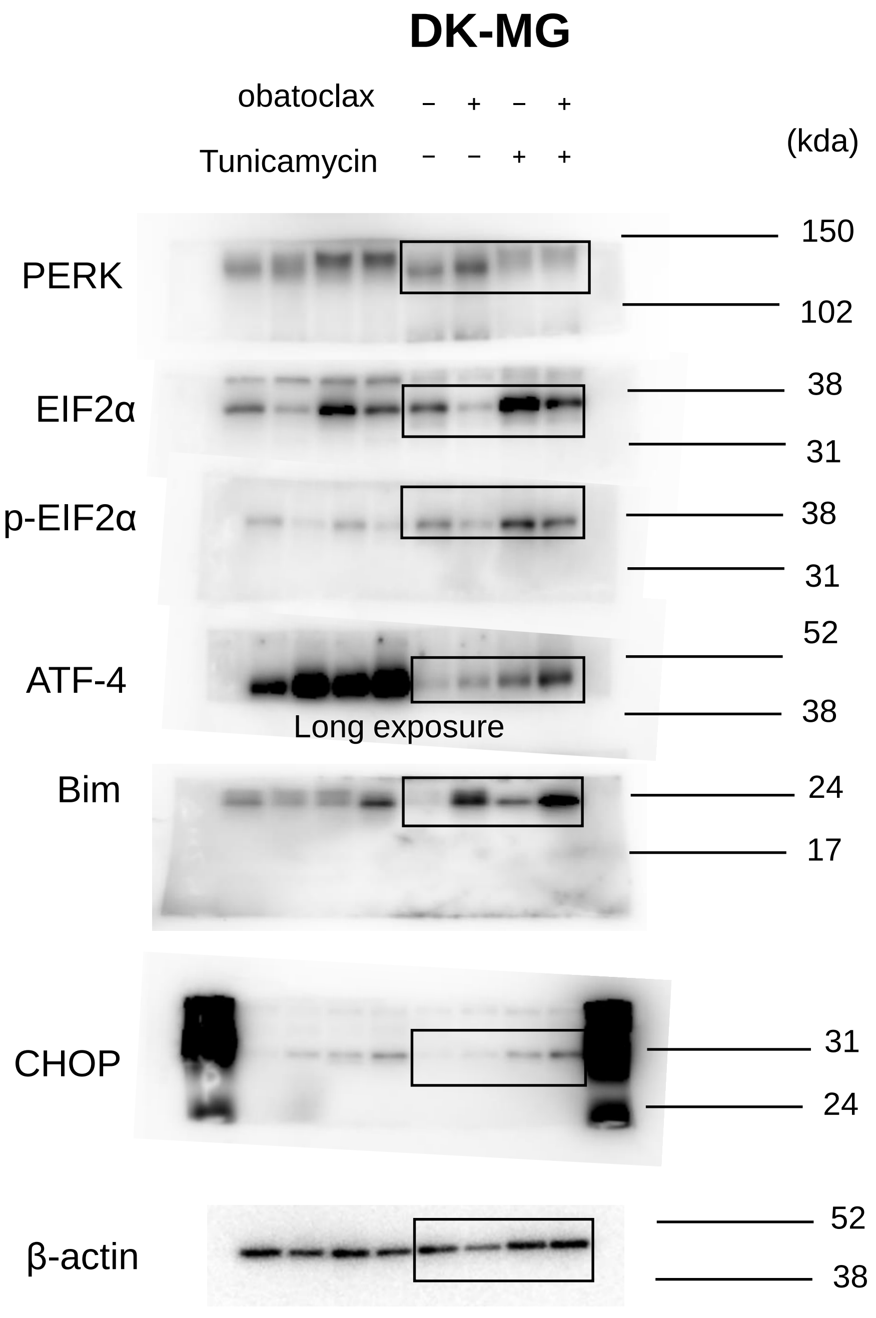

Figure 5B-1
Original Data

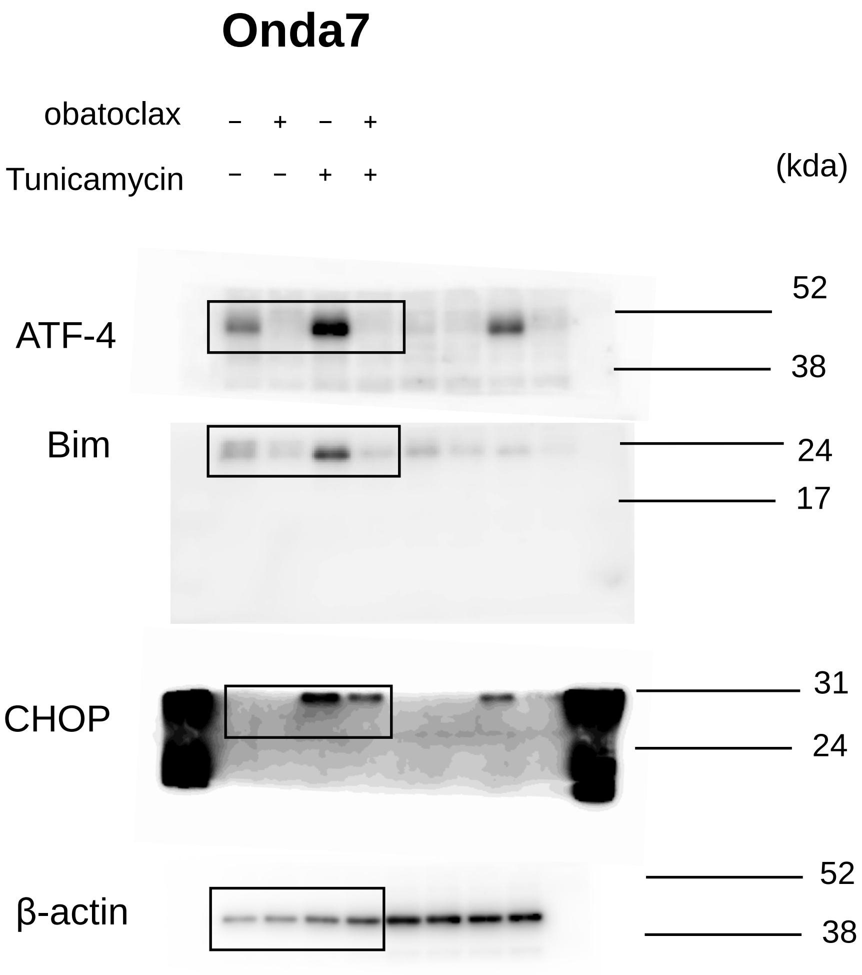

Figure 5B-2
Original Data

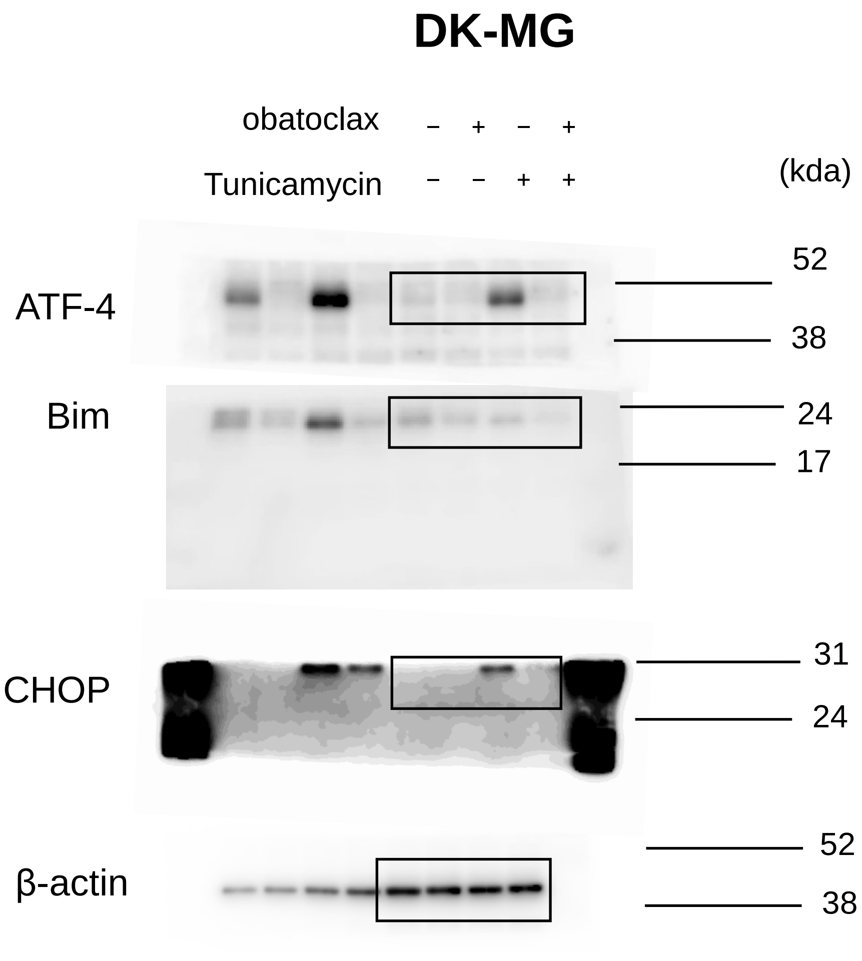

Figure 5D-1
Original Data

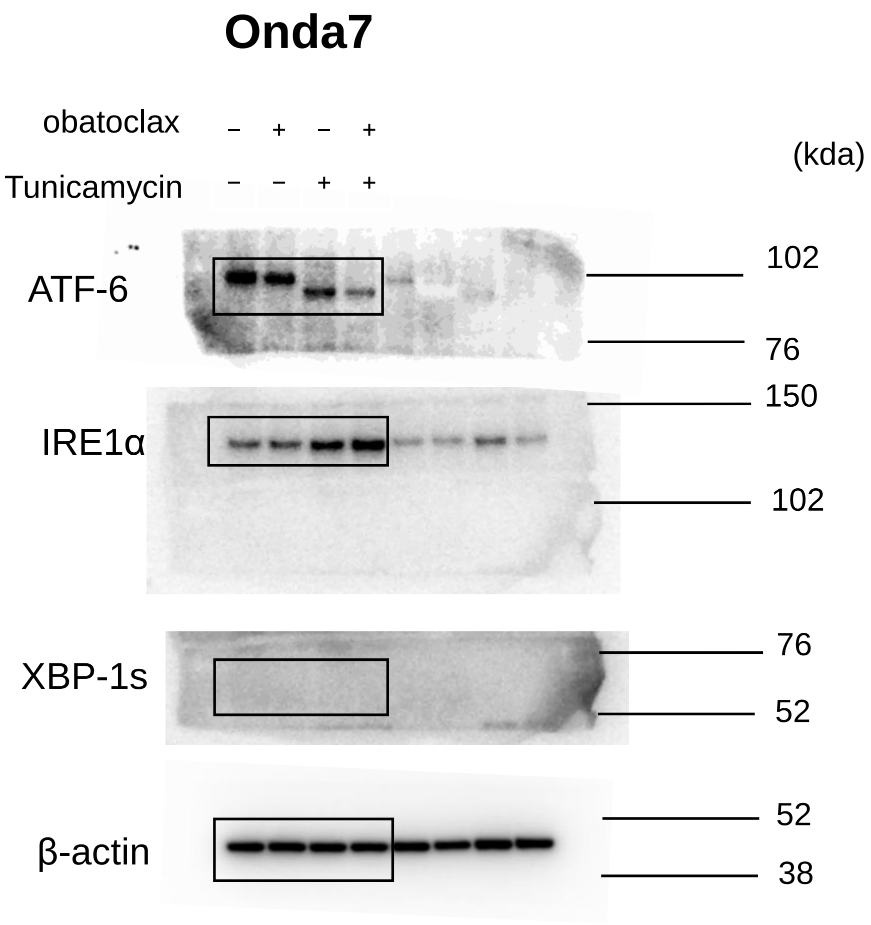

Figure 5D-2
Original Data

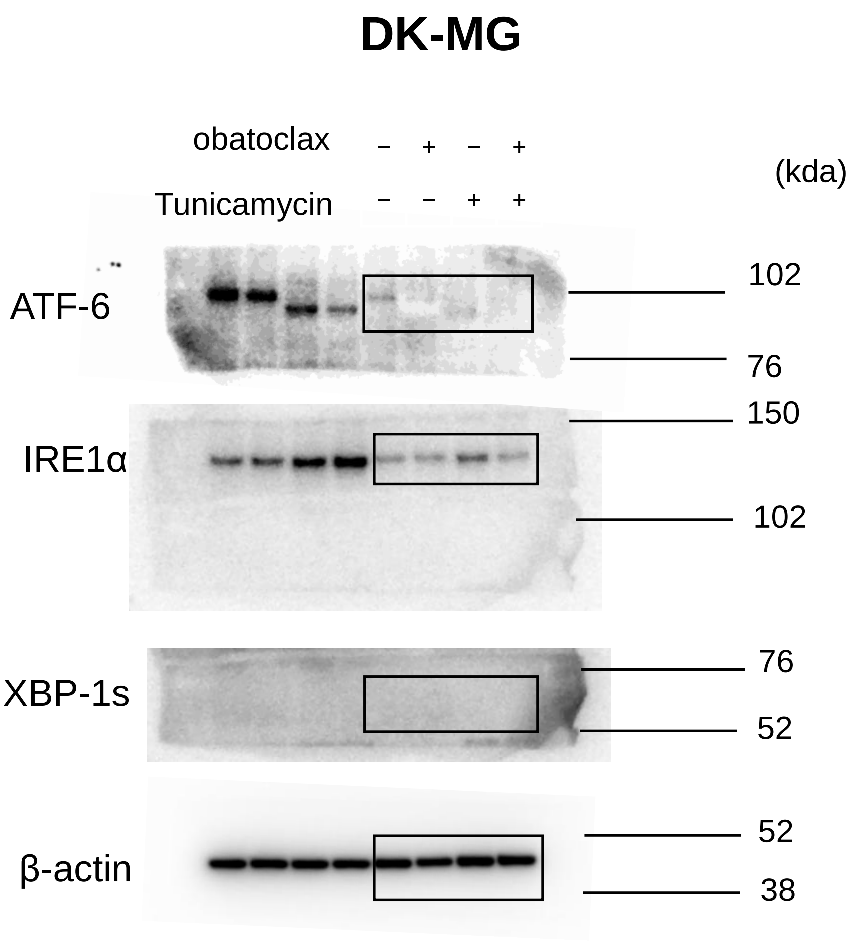

Figure 6C
Original Data

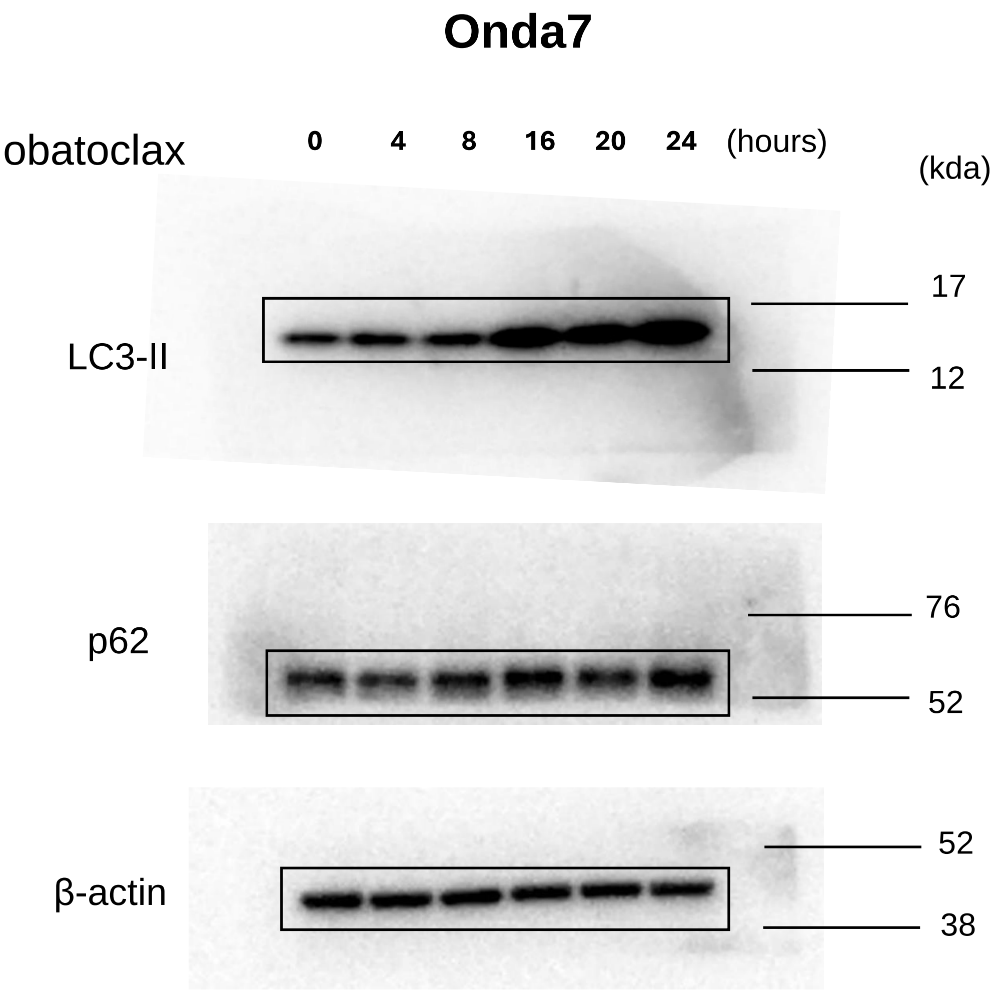

Figure 6D
Original Data

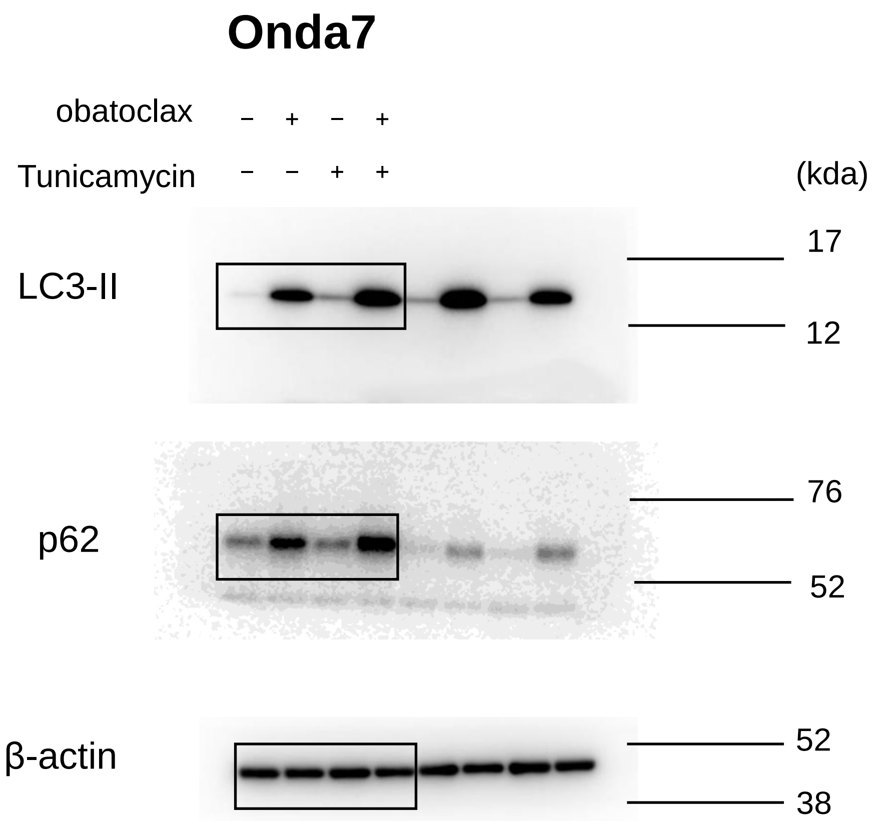

Figure 6E
Original Data

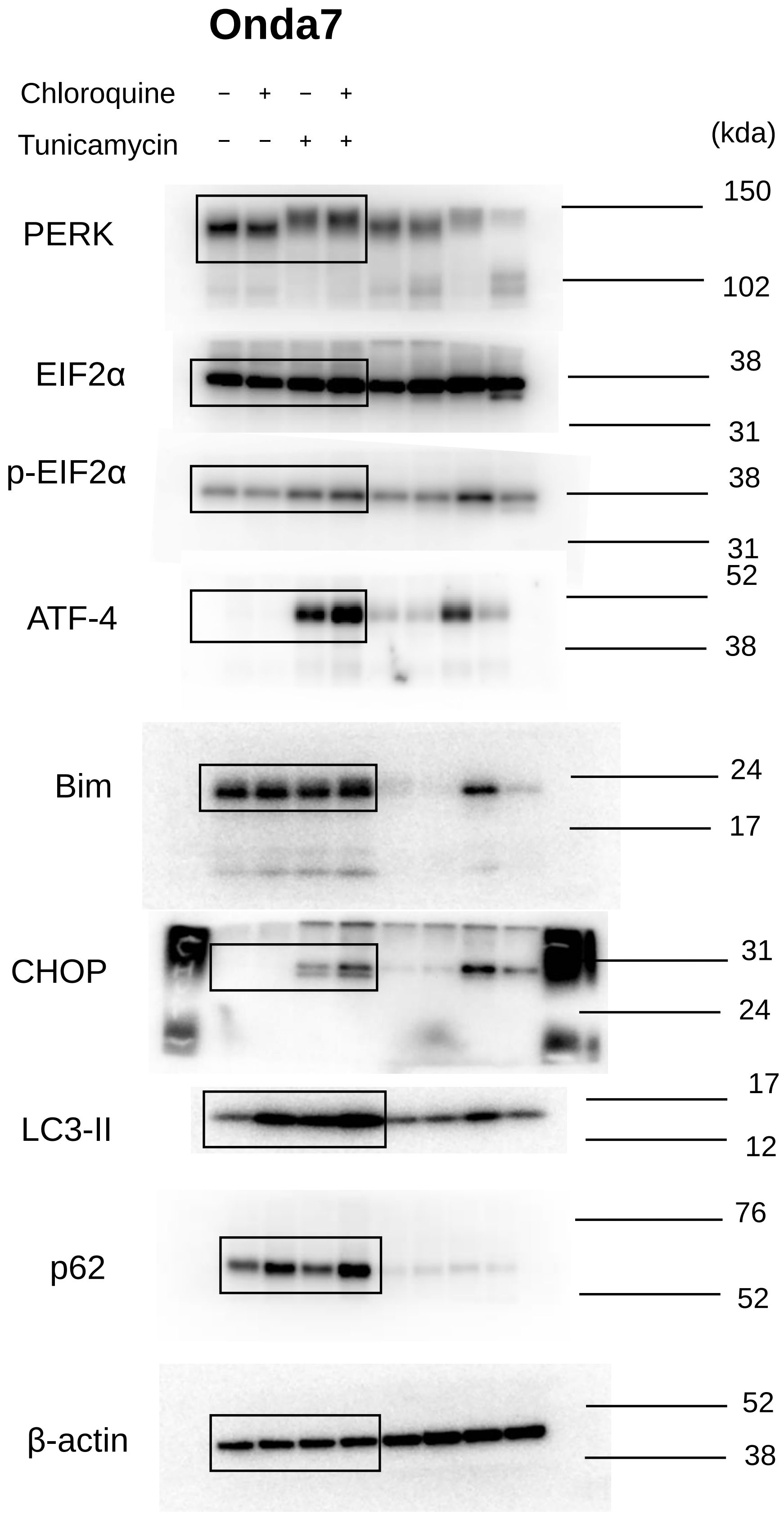

Supplementary Figure 4
Original Data

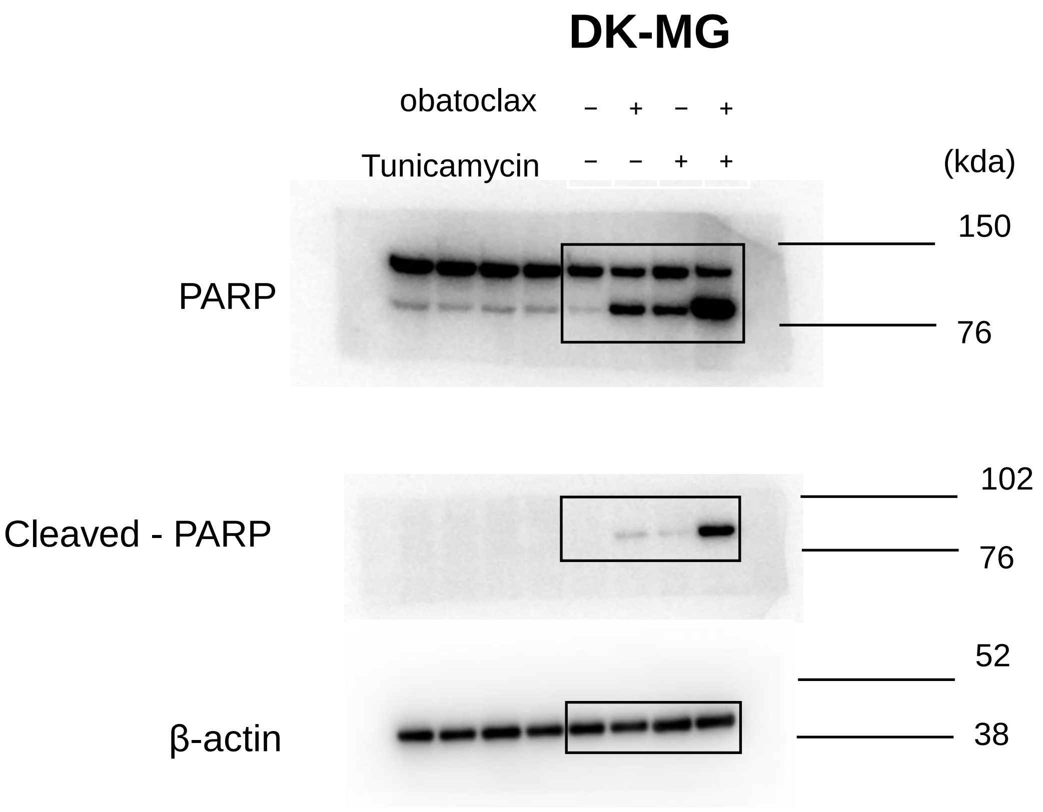

Supplementary Figure 7B
Original Data

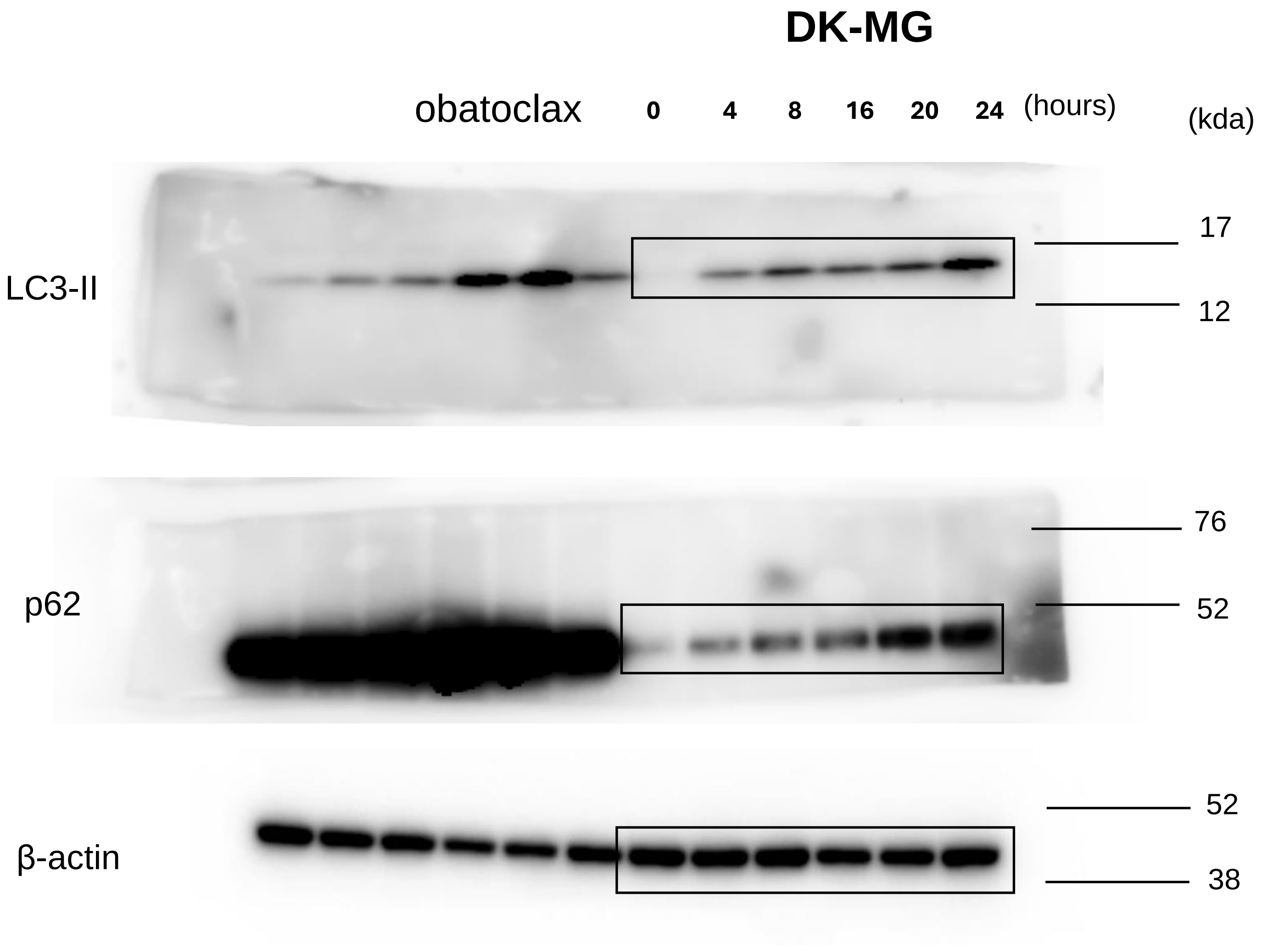

Supplementary Figure 7C
Original Data

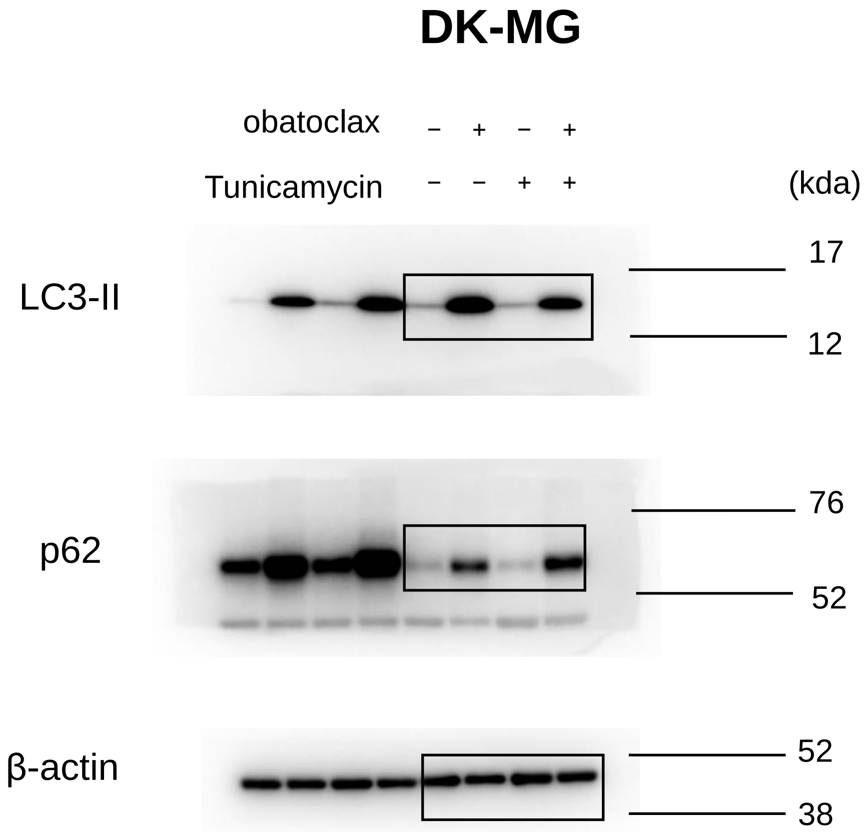

Supplementary Figure 7D
Original Data

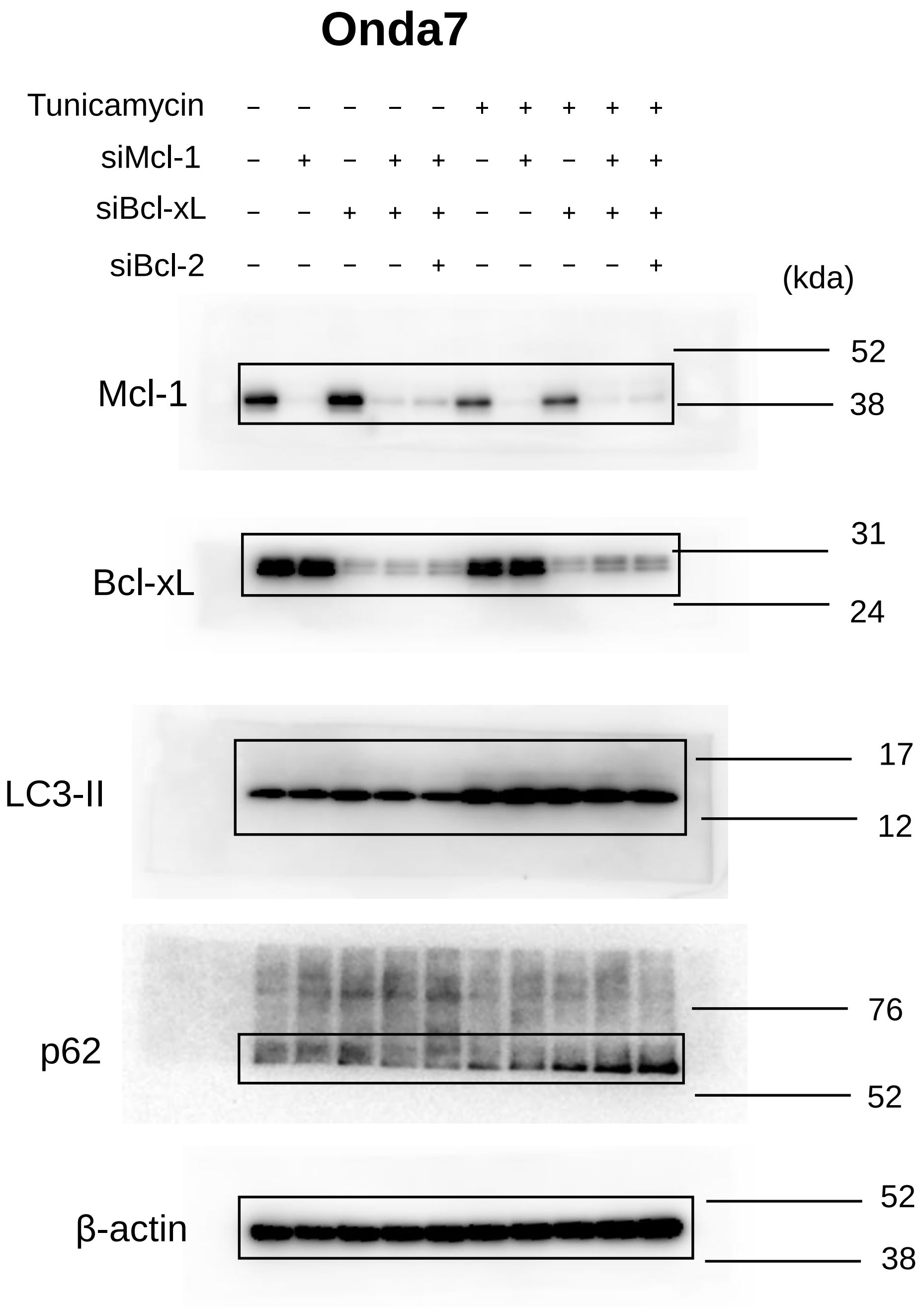

Supplementary Figure 7E
Original Data

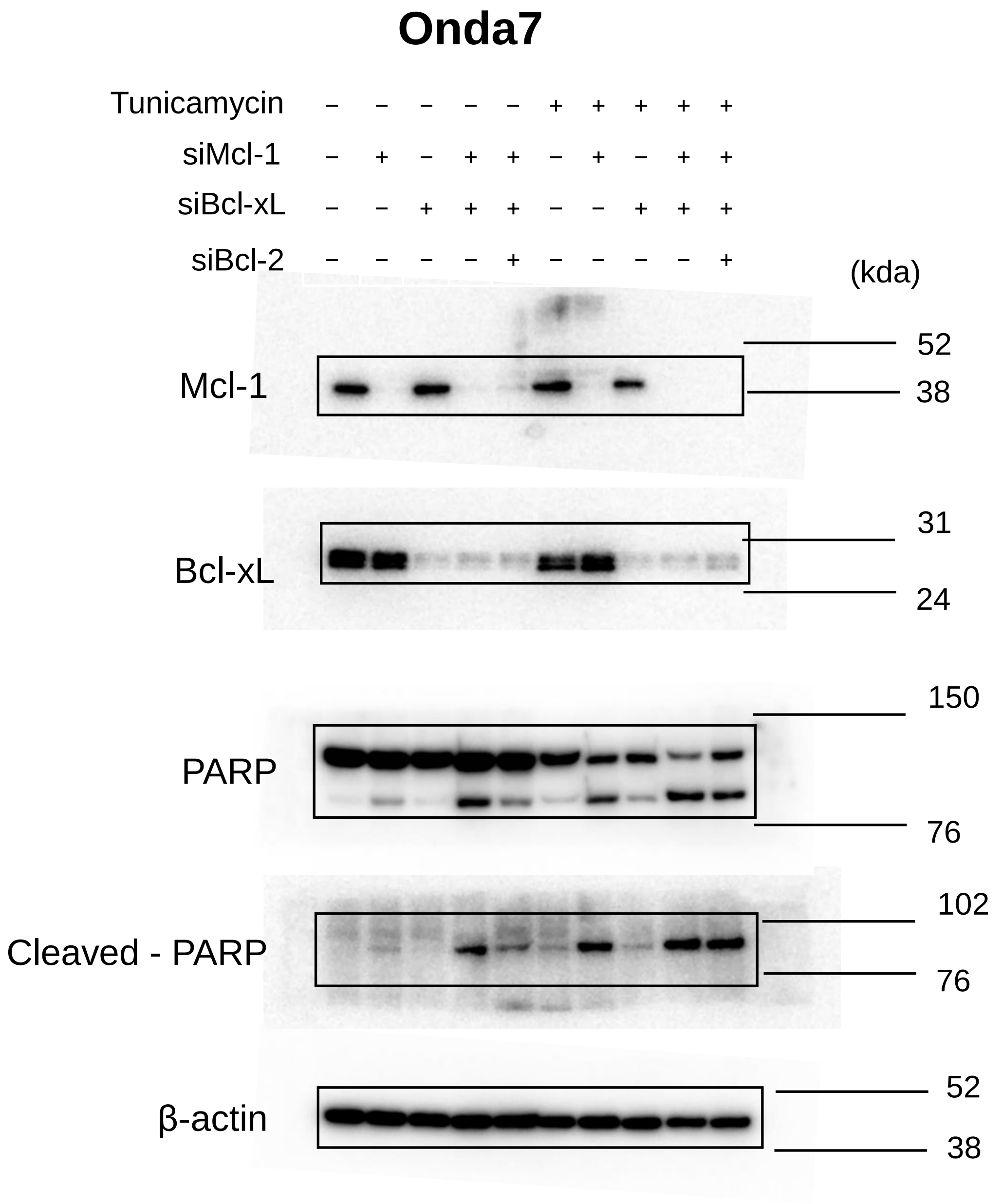

Supplement: Supplementary file 4 — Uncropped Immunoblot data [file 41420_2025_2632_MOESM4_ESM.pdf]
